# The innate immune kinase TBK1 directly increases mTORC2 activity and downstream signaling to Akt

**DOI:** 10.1016/j.jbc.2021.100942

**Published:** 2021-07-08

**Authors:** Aaron Seth Tooley, Dubek Kazyken, Cagri Bodur, Ian E. Gonzalez, Diane C. Fingar

**Affiliations:** Department of Cell and Developmental Biology, University of Michigan Medical School, Ann Arbor, Michigan, USA

**Keywords:** TBK1, mTOR, mTORC2, Akt, phosphorylation, BMDM, bone marrow–derived macrophage, EGF, epidermal growth factor, FBS, fetal bovine serum, IKKε, Iκβ kinase ε, LPS, lipopolysaccharide, mTOR, mechanistic target of rapamycin, mTORC, mTOR complex, PDGF, platelet derived growth factor, PDK1, phosphatidylinositol dependent kinase 1, PI3K, phosphatidylinositol 3′ kinase, Raptor, regulatory-associated protein of mTOR, Rictor, rapamycin-insensitive companion of mTOR, TBK1, TANK-binding kinase 1, TLR, Toll-like receptor

## Abstract

TBK1 responds to microbes to initiate cellular responses critical for host innate immune defense. We found previously that TBK1 phosphorylates mTOR (mechanistic target of rapamycin) on S2159 to increase mTOR complex 1 (mTORC1) signaling in response to the growth factor EGF and the viral dsRNA mimetic poly(I:C). mTORC1 and the less well studied mTORC2 respond to diverse cues to control cellular metabolism, proliferation, and survival. Although TBK1 has been linked to Akt phosphorylation, a direct relationship between TBK1 and mTORC2, an Akt kinase, has not been described. By studying MEFs lacking TBK1, as well as MEFs, macrophages, and mice bearing an *Mtor S2159A* knock-in allele (*Mtor*^*A/A*^) using *in vitro* kinase assays and cell-based approaches, we demonstrate here that TBK1 activates mTOR complex 2 (mTORC2) directly to increase Akt phosphorylation. We find that TBK1 and mTOR S2159 phosphorylation promotes mTOR-dependent phosphorylation of Akt in response to several growth factors and poly(I:C). Mechanistically, TBK1 coimmunoprecipitates with mTORC2 and phosphorylates mTOR S2159 within mTORC2 in cells. Kinase assays demonstrate that TBK1 and mTOR S2159 phosphorylation increase mTORC2 intrinsic catalytic activity. Growth factors failed to activate TBK1 or increase mTOR S2159 phosphorylation in MEFs. Thus, basal TBK1 activity cooperates with growth factors in parallel to increase mTORC2 (and mTORC1) signaling. Collectively, these results reveal cross talk between TBK1 and mTOR, key regulatory nodes within two major signaling networks. As TBK1 and mTOR contribute to tumorigenesis and metabolic disorders, these kinases may work together in a direct manner in a variety of physiological and pathological settings.

TANK-binding kinase 1 (TBK1) and its tissue-restricted ortholog IKKε mediate innate immunity against pathogenic viruses and bacteria in response to microbial-derived stimuli (1, 2, 3, 4, 5). Viral dsRNA and bacterial lipopolysaccharide (LPS) bind to and activate the pathogen recognition receptors Toll-like receptor 3 (TLR3) and TLR4, respectively (1, 2, 3, 4, 5). TLR3/4 activate the kinases TBK1/Iκβ kinase ε (IKKε) through phosphorylation of their activation loop site (S172) by an unknown upstream kinase or through oligomerization and activation loop site autophosphorylation (S172) (6, 7, 8, 9). TBK1/IKKε in turn phosphorylate the transcription factors IRF3 and 7 (interferon regulatory factors), resulting in their translocation into the nucleus where they induce expression of type I interferons (*e.g.*, IFNα/β), multifunctional cytokines that initiate host defense responses while limiting tissue damage (10, 11).

In prior work, we found that TBK1 phosphorylates the conserved kinase mTOR (mechanistic target of rapamycin) on S2159, which increases mTOR complex 1 (mTORC1) signaling, mTORC1-mediated cell growth (*i.e.*, cell size) and cell cycle progression, and the production of IFNβ (12, 13). This positive role for TBK1 in mTORC1 signaling has been confirmed in other studies (14, 15). While studying TBK1 and its regulation of mTORC1, we noted that cellular treatment with the TBK1/IKKε inhibitor amlexanox or TBK1 knockout in MEFs reduced phosphorylation of Akt (on S473), an important metabolic kinase and target of PI3K (13). This observation agrees with other studies (14, 15, 16, 17, 18), several of which reported that TBK1 phosphorylates Akt S473 directly (16, 17, 18). As Akt S473 represents an established target of mTOR complex 2 (mTORC2) (19, 20, 21, 22), we investigated more fully the mechanism by which TBK1 promotes Akt phosphorylation, testing the hypothesis that TBK1 directly activates mTORC2 and its downstream signaling to Akt.

mTOR comprises the catalytic kinase core of two known multisubunit mTOR complexes (mTORCs) (21, 22, 23). The scaffolding protein Raptor (regulatory-associated protein of mTOR) defines mTORC1 (24, 25), whereas the scaffolding protein Rictor (rapamycin-insensitive companion of mTOR) defines mTORC2 (26, 27). These mTORCs sense and integrate a diverse array of environmental cues to control cell physiology appropriate for cell type and context. Indeed, aberrant mTORC function contributes to pathologic states including oncogenesis and obesity-linked metabolic disorders (21, 22, 23, 28). Despite the physiological importance of mTOR, our knowledge of the upstream regulation of mTORCs remain incompletely defined, in particular mTORC2. mTORC1 drives anabolic cellular processes (*i.e.*, protein, lipid, and nucleotide synthesis) in response to the coordinated action of nutrients (*e.g.*, amino acids; glucose), growth factors (*e.g.*, EGF; IGF-1), and hormones (*e.g.*, insulin) to control cell metabolism and promote cell growth and proliferation (21, 22, 23, 29). The insulin/IGF-1 pathway represents the best-characterized activator of mTORC1, which utilizes PI3K signaling to Akt, TSC, and Rheb to activate mTORC1 on the surface of lysosomes during amino acid sufficiency (29, 30, 31). mTORC1 in turn phosphorylates a diverse set of targets (32, 33), with S6K1 T389 phosphorylation serving as a widely employed readout of mTORC1 activity in intact cells.

Growth factors and hormones also activate mTORC2 in a manner that requires PI3K (21, 22, 23, 31, 34). It is important to note that the upstream regulation of mTORC2 by diverse inputs remains significantly less well defined than mTORC1. Recently, we demonstrated that the energy sensing kinase AMPK activates mTORC2 directly to promote cell survival during energetic stress (35). In addition, the stress-inducible protein Sestrin2, which protects against stress injury and improves homeostasis, was shown to activate mTORC2 (36). mTORC2 in turn phosphorylates Akt S473, a widely employed readout of mTORC2 activity in intact cells (19, 20, 22). Akt functions as a key mediator of PI3K signaling that controls diverse aspects of cell physiology, including cell metabolism and survival (31, 37). Akt activation absolutely requires phosphorylation of its activation loop site (T308) by phosphatidylinositol dependent kinase 1 (PDK1). Phosphorylation of its hydrophobic motif site (S473) by mTORC2 activates Akt further to a maximal level and controls substrate preference (38). In some contexts, Akt S473 phosphorylation promotes and/or stabilizes Akt T308 phosphorylation, as increases or decreases in Akt P-S473 often result in correspondingly similar changes in Akt P-T308 (19, 35, 39). It is important to note that, in addition to mTORC2, several other kinases have been suggested as Akt S473 kinases including DNA-PK, ATM, and ILK, and more recently TBK1 and IKKε (16, 17, 18, 40). Functionally, mTORC2 controls cell metabolism, modulates the actin cytoskeleton, and promotes cell survival (21, 22, 23, 26, 27, 41).

Beyond its well-known role in innate immunity, TBK1 has been implicated in oncogenesis and metabolic disorders linked to obesity such as type II diabetes, similar to mTOR and Akt (14, 15, 16, 17, 28, 42, 43, 44, 45, 46, 47, 48, 49, 50, 51, 52, 53, 54). In oncogenic KRas-transformed cells, TBK1 promotes cell proliferation and survival and the growth of tumor explants *in vivo*, with either mTORC1 or Akt suggested as downstream mediators of TBK1 action (14, 15, 16, 17, 42, 43, 44, 45, 46). In addition, elevated expression of the TBK1 ortholog IKKε contributes to breast cancer oncogenesis (44, 55). In mice with diet-induced obesity, adipocyte-specific knockout of TBK1 decreases Akt S473 phosphorylation in white adipose tissue, increases whole-body insulin resistance and proinflammation, and impairs glucose homeostasis (50, 54), a phenotype that overlaps with that resulting from adipocyte-specific knockout of Raptor (mTORC1) or Rictor (mTORC2) (28, 56, 57, 58). Moreover, treatment of obese mice or human patients with amlexanox, or knockout of TBK1, Raptor (mTORC1 partner protein), or S6K1 (mTORC1 substrate) in mouse adipocytes, reduces adiposity and body mass, in part due to increased energy expenditure (50, 52, 54, 59).

To better understand how TBK1 contributes to health and disease, we investigated the molecular mechanism by which TBK1 promotes the phosphorylation of Akt. We find that TBK1 phosphorylates mTOR to activate mTORC2 directly, resulting in increased Akt phosphorylation during cellular treatment with EGF and other growth factors. Moreover, by studying *Mtor S2159A* knock-in mice (*i.e.*, *Mtor*^*A/A*^ mice) and primary macrophages from these mice, we find that TBK1-mediated mTOR phosphorylation increases mTORC2 signaling to Akt in primary macrophages and *in vivo* in response to the TLR3-activating agonist poly(I:C), which mimics viral dsRNA. This work not only elucidates the poorly defined upstream activation of mTORC2 but also improves our understanding of the contribution of TBK1 and mTORCs to physiology and pathologic conditions such as tumorigenesis and obesity-linked metabolic disorders.

## Results

### TBK1 increases mTOR-dependent Akt phosphorylation in response to EGF

**Figure 1:**
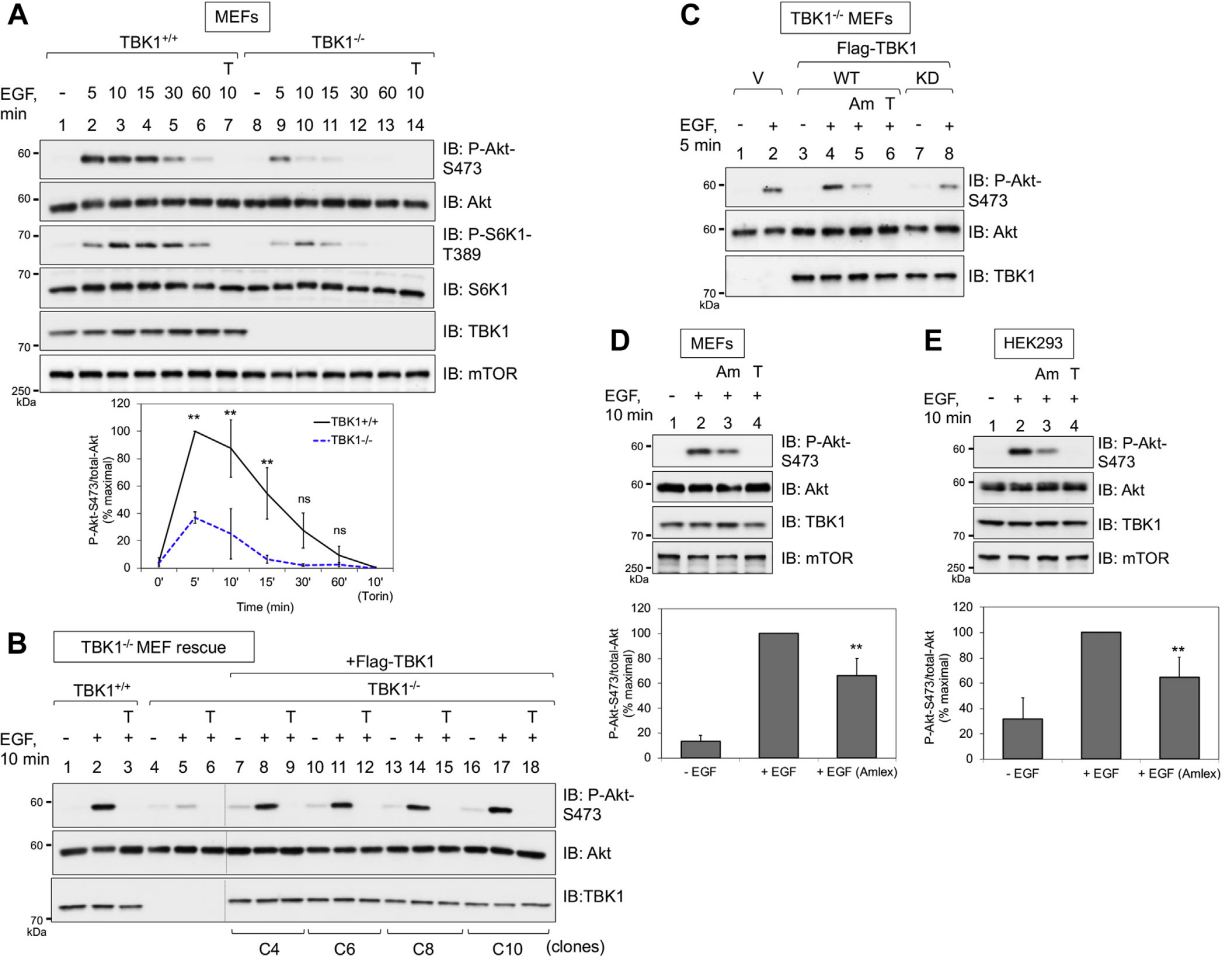
**TBK1 promotes mTOR-dependent Akt phosphorylation in response to EGF.***A*, reduced EGF stimulated Akt S473 phosphorylation in TBK1 null MEFs. TBK1^+/+^ and TBK1^−/−^ MEFs were serum starved overnight (20 h), pretreated with Torin1 (T) (100 nM, 30 min), and stimulated without (−) or with EGF (50 ng/ml) for the indicated times (in minutes, min). Whole-cell lysates (WCLs) were immunoblotted with the indicated antibodies. *Graph*, quantification of results. Mean ratio ± SD of Akt P-S473 over total-Akt from three independent experiments, normalized as percent of maximal (+EGF 5 min in TBK1^+/+^ MEFs set to 100%). Statistical significance was measured using paired Student’s *t* test (assuming equal variances). ∗∗*p* < 0.01; “ns”, not significant. *B*, rescue of TBK1 null MEFs with Flag-TBK1. TBK1^+/+^ MEFs, TBK1^−/−^ MEFs, and clones of TBK1^−/−^ MEFs stably expressing Flag-TBK1 were serum starved, pretreated with Torin1 (100 nM, 30 min), and stimulated without (−) or with (+) EGF (50 ng/ml) for 10 min. WCLs were immunoblotted with the indicated antibodies. *C*, rescue of TBK1 null MEFs with wildtype *versus* kinase dead Flag-TBK1. Pools of drug-resistant TBK1^−/−^ MEFs stably expressing wildtype or kinase dead (K38M) Flag-TBK1 were analyzed as in *A* and *B*. *D*, amlexanox reduces EGF-stimulated mTORC2 signaling in MEFs. TBK1^+/+^ MEFs were serum starved overnight (20 h), pretreated with amlexanox (Am) (100 μM, 2 h) or Torin1 (T) (100 nM, 30 min), and stimulated with EGF as in 1A. Whole-cell lysates were immunoblotted with the indicated antibodies. *Graph*, quantification of results. Mean ratio ± SD of Akt P-S473 over total-Akt from five independent experiments, normalized as percent of maximal (+EGF 10 min set to 100%). Statistical significance was measured using paired Student’s *t* test (assuming equal variances). ∗∗*p* < 0.01 relative to TBK1^+/+^ MEFs stimulated +EGF in the absence of amlexanox. *E*, effect of amlexanox on EGF-stimulated mTORC2 signaling in HEK293 cells. HEK293 cells were analyzed as in *D*. *Graph*, quantification of results. Mean ratio ± SEM of Akt P-S473 over total-Akt was calculated from five independent experiments as in *D*. All experiments were performed three times or more.

To elucidate the mechanism by which TBK1 positively controls Akt phosphorylation, we first analyzed TBK1 wildtype (TBK1^+/+^) and knockout (TBK1^−/−^) MEFs. TBK1^−/−^ MEFs displayed significantly reduced phosphorylation of Akt S473 (Fig. 1*A*) across an EGF time course, consistent with prior work (13, 16, 17). TBK1^−/−^ MEFs also displayed reduced Akt T308 phosphorylation in response to EGF (Fig. S1), and the active-site mTOR inhibitors Torin1 and Ku-0063794 ablated both Akt P-S473 and P-T308 (Fig. 1*A* and Fig. S1). These data are consistent with mTORC2 functioning as a major Akt S473 kinase (19, 20) and with many reports that Akt S473 phosphorylation promotes and/or stabilizes Akt T308 phosphorylation (19, 35, 39). Consistent with our prior work (13), TBK1^−/−^ MEFs displayed reduced S6K1 T389 phosphorylation, confirming that TBK1 promotes mTORC1 signaling (Fig. 1*A*). To confirm that reduced EGF-stimulated Akt phosphorylation in TBK1^−/−^ MEFs results from loss of TBK1, we stably expressed vector control or Flag-tagged TBK1 in TBK1^−/−^ MEFs by lentiviral transduction followed by puromycin selection. We selected several independent clones in which expression of exogenous Flag-TBK1 matched endogenous TBK1, as our prior work found that overexpression of TBK1 functions in a dominant negative manner to inhibit mTORC1 signaling (13), similar to overexpression of the mTORC1 subunit Raptor, an artifact common for proteins with scaffolding function. Expression of Flag-TBK1 rescued the reduced Akt S473 phosphorylation displayed in TBK1^−/−^ MEFs stimulated with EGF (Fig. 1*B*). Moreover, stable expression of kinase-dead Flag-TBK1 failed to rescue P-Akt S473 (Fig. 1*C*). These results indicate that the kinase activity of TBK1 rather than its scaffolding function promotes Akt phosphorylation. Consistent with this conclusion, the TBK1/IKKε inhibitor amlexanox significantly reduced Akt P-S473 in response to EGF in MEFs (Fig. 1*D*) and HEK293 cells (Fig. 1*E*). Taken together, these results indicate that TBK1 kinase activity promotes mTOR-dependent phosphorylation of Akt S473 and T308 during EGF stimulation.

### Physiological levels of TBK1 expression increase Akt S473 phosphorylation through mTOR

**Figure 2:**
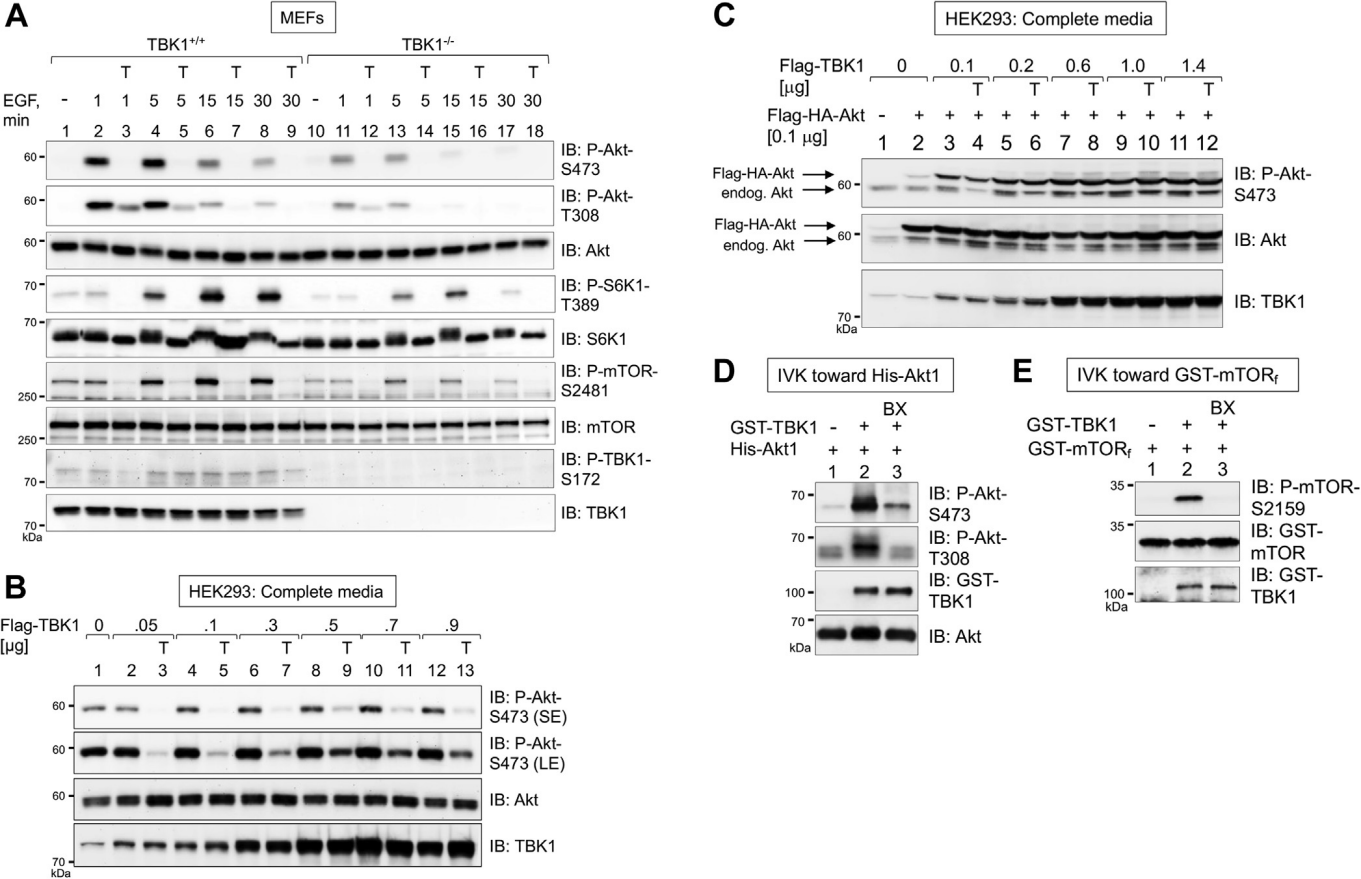
**Physiological levels of TBK1 expression increase Akt S473 phosphorylation through mTOR.***A*, Torin1 reduces EGF-stimulated Akt S473 phosphorylation. TBK1^+/+^ and TBK1^−/−^ MEFs were serum starved overnight (20 h), pretreated with Torin1 (T) (100 nM, 30 min), and stimulated without (−) or with EGF (50 ng/ml) for the times indicated (in minutes, min). Whole-cell lysates (WCLs) were immunoblotted with the indicated antibodies. *B*, TBK1 overexpression increases Akt P-S473 in a Torin1-sensitive manner. HEK293 cells were transfected with increasing amounts of Flag-TBK1 (0–0.9 μg per 60 mm plate) in duplicate. Approximately 24 h post transfection, cells in complete media were treated with Torin1 (T) (100 nM; 30 min). WCLs were immunoblotted with the indicated antibodies. *C*, TBK1 and Akt co-overexpression increases Akt S473 phosphorylation in a largely Torin1-insensitive manner. HEK293 cells were cotransfected with increasing amounts of Flag-TBK1 (0–1.4 μg per 60-mm plate) together with a constant amount of Flag-HA-Akt (0.1 μg) in duplicate. Cells were treated with Torin1 and analyzed as in Figure 1*B*. *D*, recombinant TBK1 phosphorylates His-Akt1 *in vitro*. Recombinant, active GST-TBK1 (50 ng) and His-Akt1 (50 ng) were incubated together in an *in vitro* kinase (IVK) reaction with ATP at 30 °C for 30 min, as indicated. The IVK reaction in lane 3 included pretreatment with BX-795 (BX) (10 μM) for 30 min prior to initiation of the reaction with ATP. IVK reactions were immunoblotted with the indicated antibodies. *E*, recombinant TBK1 phosphorylates GST-mTOR_f_*in vitro*. GST-TBK1 (100 ng) was incubated with GST-mTOR_f_ (50 ng) at 30 °C for 30 min, as indicated. As in *D*, the IVK reaction in lane 3 included pretreatment with BX-795 (BX) (15 μM). IVK reactions were immunoblotted with the indicated antibodies. All experiments were performed three times or more. LE, long exposure; SE, short exposure.

Subsequent to the identification of mTORC2 as the major Akt S473 kinase (19, 20, 22), several groups demonstrated that TBK1 and IKKε phosphorylate Akt S473 and T308 directly *in vitro* (16, 17, 18). As we published previously that TBK1 phosphorylates mTOR to increase mTORC1 activity and signaling (13), we sought to clarify roles for TBK1 *versus* mTOR in phosphorylation of Akt. We therefore assessed effects of mTOR inhibition across an EGF time course. As in Figure 1, TBK1 knockout reduced Akt S473 phosphorylation at each time point (1–30 min) following EGF stimulation (Fig. 2*A*). TBK1 knockout also reduced Akt T308 phosphorylation. Pretreatment with Torin1 ablated Akt P-S473 and P-T308 at each time point in response to EGF in both TBK1^+/+^ and TBK1^−/−^ MEFs (Fig. 2*A*). Consistent with our prior work, TBK1 knockout reduced S6K1 P-T389 (*i.e.*, mTORC1 signaling) and mTOR autophosphorylation on S2481 (13). It is important to note that mTOR S2481 autophosphorylation represents a simple method to monitor total mTOR- or mTORC-specific catalytic activity in intact cells (60). These results indicate that, in this setting, TBK1 increases Akt S473 and T308 phosphorylation in response to EGF in a manner that requires mTOR activity.

We next investigated whether elevated levels of TBK1 and/or Akt enables TBK1 to directly engage and phosphorylate Akt. We therefore overexpressed increasing amounts of Flag-TBK1 in HEK293 cells. Consistent with prior work (16, 17, 18), exogenous Flag-TBK1 WT increased Akt P-S473 (Fig. 2*B*). Torin1 reduced Akt P-S473 at low to mid doses of Flag-TBK1, indicating mTOR dependency, whereas higher doses displayed less dependence on mTOR activity. We next cotransfected Flag-TBK1 together with Flag-HA-Akt. The double Flag-HA tag allowed resolution of exogenous Akt (Flag-HA tagged) (upper band) from endogenous Akt (lower band), enabling distinct assessment of phosphorylation on each of these two Akt populations. As before, TBK1 overexpression increased phosphorylation of endogenous Akt in a Torin1-sensitive manner at low doses. Upon coexpression of Flag-TBK1 with Flag-HA-Akt, however, Akt P-S473 became Torin1 resistant at even the lowest dose of Flag-TBK1 (Fig. 2*C*). These data indicate that, in the context of TBK1 and Akt overexpression, TBK1 phosphorylates Akt S473 independently of mTOR activity, as reported in prior work (16, 17). At physiological levels of protein, however, TBK1 requires mTOR activity to mediate Akt S473 phosphorylation. By *in vitro* kinase assay, we confirmed that recombinant active TBK1 phosphorylates His-Akt1 on S473 and T308 (Fig. 2*D*), consistent with other groups (16, 17, 18), and that recombinant TBK1 phosphorylates a GST-mTOR fragment on S2159 (Fig. 2*E*), consistent with our prior work (13). Phosphorylation of both His-Akt1 and GST-mTOR_f_ by recombinant TBK1 were sensitive to BX-795, a TBK1/IKKε inhibitor (Fig. 2, *D* and *E*). Thus, TBK1 phosphorylates diverse substrates with dissimilar consensus phosphorylation motifs, at least *in vitro*, particularly at elevated levels of expression of kinase and/or substrate.

**Figure 3:**
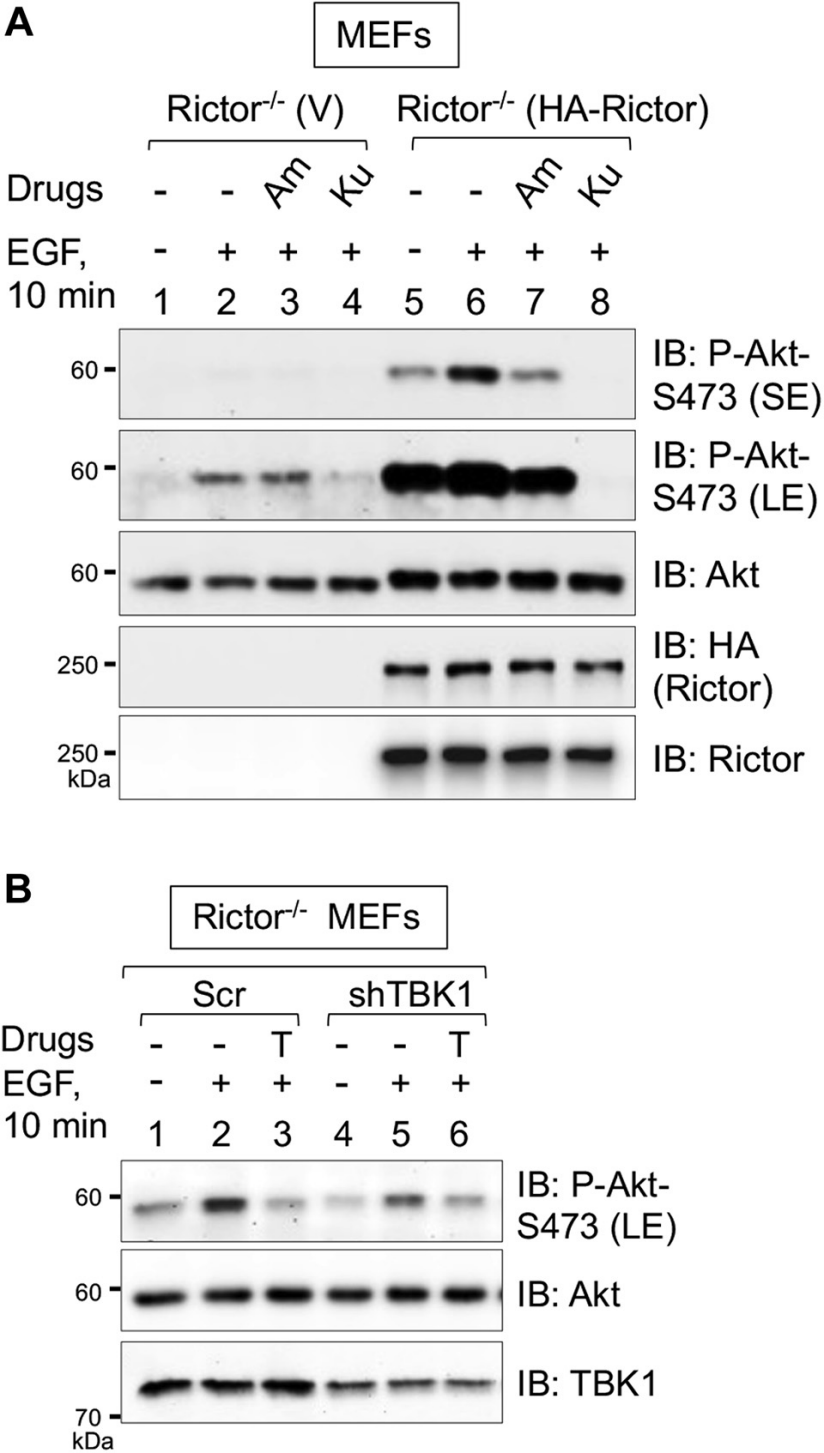
**Rictor null MEFs retain a low level of mTOR-dependent Akt S473 phosphorylation supported by TBK1 expression but not TBK1 activity.***A*, Ku-0063794 (Ku) but not amlexanox reduces Akt S473 phosphorylation in Rictor null MEFs. Rictor^−/−^ MEFs rescued with vector control (V) or HA-Rictor were serum starved overnight (20 h), pretreated with amlexanox (Am) (100 μM, 2 h) or Ku-0063794 (Ku) (100 nM, 30 min), and stimulated without (−) or with (+) EGF (50 ng/ml) for 10 min. Whole-cell lysates (WCLs) were immunoblotted with the indicated antibodies. n = 3 experiments. *B*, TBK1 knockdown reduces Akt S473 phosphorylation in Rictor null MEFs. Rictor^−/−^ MEFs were transduced with lentiviral particles encoding scrambled (Scr) shRNA or an shRNA targeting TBK1 and selected in puromycin. The MEFs were then serum starved overnight (20 h), pretreated with Torin1 (T) (100 nM, 30 min), and stimulated without (−) or with (+) EGF (50 ng/ml) for 10 min. WCLs were immunoblotted with the indicated antibodies. n = 2 experiments. LE, long exposure; SE, short exposure.

To examine a potential role for TBK1 in the phosphorylation of Akt S473 in the absence of confounding mTORC2 activity, we analyzed MEFs lacking the critical mTORC2 partner protein Rictor. As expected, Rictor^−/−^ MEFs expressing vector control displayed extremely low Akt S473 phosphorylation in response to EGF, while rescue with HA-Rictor rescued this phenotype in a manner sensitive to the mTOR inhibitor Ku-0063794 (Fig. 3*A*). Consistent with Figure 1, *D* and *E*, amlexanox reduced Akt P-S473 in the rescued Rictor^−/−^ MEFs, indicating dependence on TBK1 activity (Fig. 3*A*). Upon long blot exposure time, however, EGF increased Akt P-S473 in the Rictor^−/−^ MEFs in a Ku-0063794-sensitive but amlexanox-resistant manner (Fig. 3*A*). Although somewhat unexpected, this Ku-0063794 sensitivity suggests that MEFs lacking Rictor, and thus expressing crippled mTORC2, still retain a low level of activity toward Akt S473. In agreement, Xie *et al.* (17) found that mTOR inhibition with Torin1 reduced Akt P-S473 in response to platelet-derived growth factor (PDGF) in Rictor^−/−^ MEFs. Therefore, these data indicate that mTOR rather than another kinase (*e.g.*, TBK1) mediates Akt S473 phosphorylation in Rictor^−/−^ MEFs. The amlexanox resistance of Rictor^−/−^ MEFs suggests that TBK1 activity contributes negligibly to Akt phosphorylation in the context of crippled mTORC2. Curiously, shRNA-mediated knockdown of TBK1 in Rictor^−/−^ MEFs reduced Akt P-S473 (Fig. 3*B*), consistent with Xie *et al.* (17). This finding suggests that the scaffolding function of TBK1 may contribute to mTORC2-mediated phosphorylation of Akt S473, at least in cells lacking physiologically intact mTORC2. Finally, it is important to note that our prior work demonstrated that Rictor^−/−^ MEFs possess intact, TBK1-dependent S6K1 T389 phosphorylation in response to EGF (13). Thus, activation of mTORC1 by TBK1 occurs independently of TBK1-mTORC2. As mTORC2-mediated phosphorylation of S473 modulates Akt signaling to mTORC1 in certain contexts (due to effects of Akt P-S473 on Akt P-T308), it remains possible that, in certain settings, TBK1-mediated activation of mTORC2 may influence mTORC1 signaling. Taken together, these data indicate that mTORC2 represents a critical link between TBK1 and Akt S473 phosphorylation at physiological levels of TBK1 and Akt protein expression.

### mTOR S2159 phosphorylation promotes mTORC2 signaling in response to EGF

**Figure 4:**
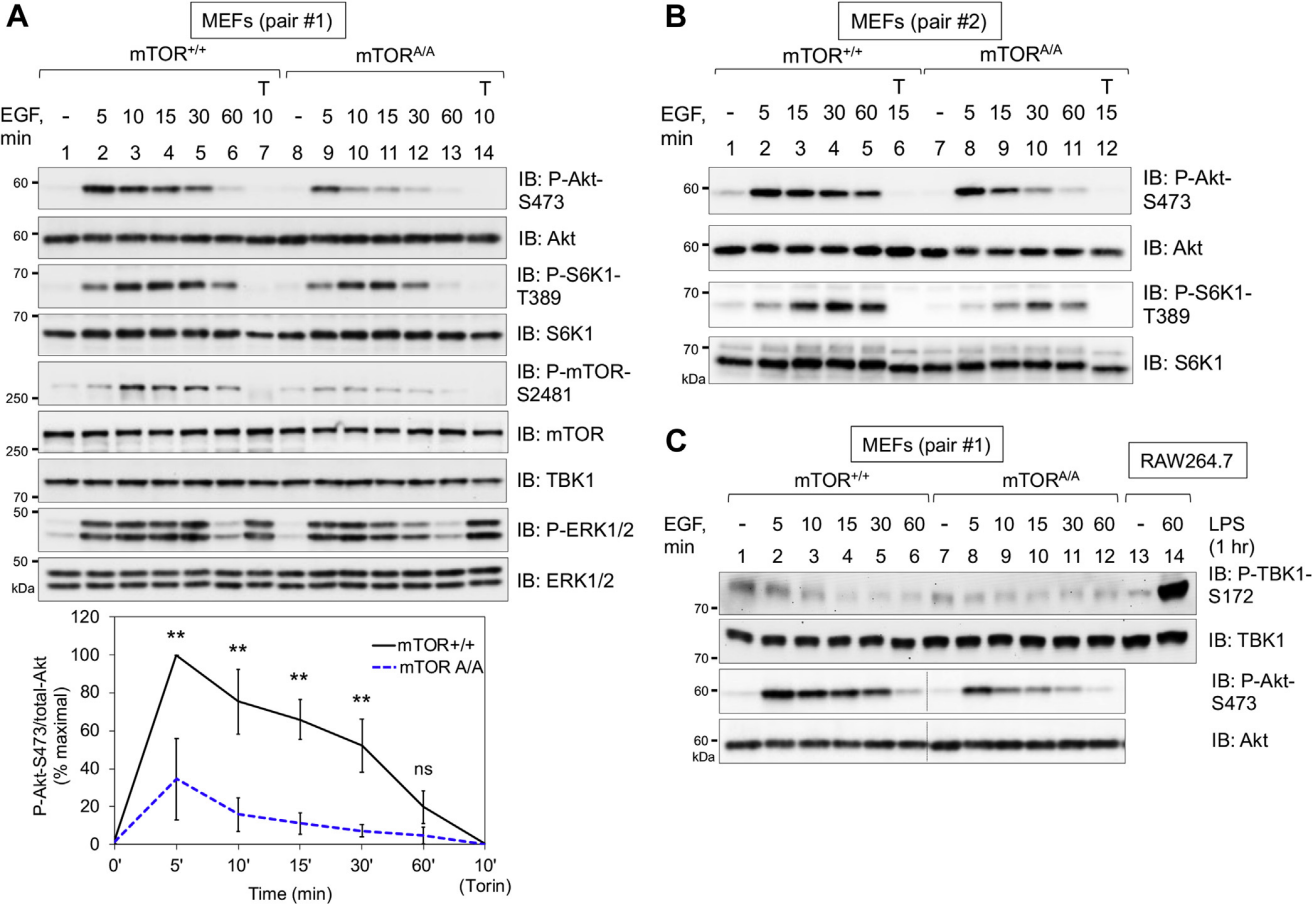
**mTOR S2159 phosphorylation promotes mTORC2 signaling in response to EGF.***A*, mTOR S2159A knock-in reduces mTORC2 signaling. Immortalized mTOR^+/+^ and mTOR^A/A^ MEFs (pair #1) were serum starved overnight (20 h), pretreated with Torin1 (T) (100 nM, 30 min), and stimulated without (−) or with EGF (50 ng/ml) for the times indicated (in minutes, min). Whole-cell lysates (WCLs) were immunoblotted with the indicated antibodies. *Graph*, quantification of results. Mean ratio ± SD of Akt P-S473 over total-Akt from four independent experiments, normalized as percent of maximal (+EGF 5 min in mTOR^+/+^ MEFs set to 100%). Statistical significance of differences was measured using paired Student’s *t* test (assuming equal variances). ∗∗*p* < 0.01; “ns”, not significant. *B*, effect of EGF on Akt P-S473 in a second pair of immortalized wildtype and mTOR S2159A knock-in MEFs. Immortalized mTOR^+/+^ and mTOR^A/A^ MEFs (pair #2) were treated as in *A*. *C*, EGF does not increase TBK1 activation loop phosphorylation (S172) in mTOR^+/+^ or mTOR ^A/A^ MEFs. mTOR^+/+^ and mTOR^A/A^ MEFs (pair #1) were serum starved and stimulated with EGF for the times indicated, as in *A*. RAW264.7 macrophages in complete media were stimulated without (−) or with (+) LPS (100 ng/ml, 60 min) to serve as a positive control for TBK1 P-S172 Western blotting. WCLs from mTOR MEFs and RAW264.7 macrophages were resolved on the same gel and immunoblotted with the indicated antibodies. All experiments were performed three times or more.

In prior work we generated genome-edited mice bearing an alanine knock-in substitution at *Mtor S2159* using CRISPR-Cas9 technology (13). By studying bone marrow–derived macrophages (BMDMs) in culture isolated from wildtype (*Mtor*^*+/+*^) and S2159A knock-in mice (*Mtor*^*A/A*^), we demonstrated that mTOR S2159 phosphorylation is required for mTORC1 signaling and IFNβ production in macrophages stimulated with innate immune agonists (*i.e.*, poly(I:C); LPS) (13). Therefore, we next investigated a potential direct link between TBK1 and mTORC2 by studying the role of mTOR S2159 phosphorylation in control of mTORC2 signaling. To do so, we isolated littermate-matched MEFs from *Mtor*^+/+^ and *Mtor*^A/A^ mice (pair #1 MEFs), subjected them to spontaneous immortalization, and analyzed their response to EGF following serum deprivation. Relative to MEFs from *Mtor*^*+/+*^ mice (*i.e.*, mTOR^+/+^ MEFs), MEFs from *Mtor*^*A/A*^ mice (*i.e.*, mTOR^A/A^ MEFs) displayed significantly reduced Akt P-S473 (Fig. 4*A*) and Akt P-T308 (Fig. S2*A*) across an EGF time course. mTOR^A/A^ MEFs also displayed reduced S6K1 P-T389 and mTOR S2481 autophosphorylation (Fig. 4*A*). Activation of the MAPK/ERK pathway in response to EGF remained unperturbed in the mTOR^A/A^ MEFs (as monitored by the phosphorylation of ERK1/2 on T202/Y204), indicating intact activation of EGF-receptor signaling to MAPK/ERK in mTOR^A/A^ MEFs (Fig. 4*A*). Of importance, we observed reduced Akt P-S473 and S6K1 P-T389 in response to EGF in a second, independently derived pair of mTOR^+/+^ and mTOR^A/A^ MEFs (pair #2 MEFs) (Fig. 4*B*). These data demonstrate that mTOR S2159 phosphorylation increases mTOR catalytic activity and promotes mTORC2 and 1 signaling upon cellular stimulation with EGF.

We next asked whether EGF activates TBK1 by monitoring TBK1 phosphorylation on its activation loop site (S172). We found that EGF failed to increase TBK1 P-S172 in either mTOR MEFs (+/+ and A/A) (Fig. 4*C*) or TBK1 MEFs (+/+ and −/−) (Fig. S2*B*). We analyzed LPS treatment of RAW264.7 macrophages as a positive control. As expected, LPS strongly increased TBK1 P-S172 (Fig. 4*C*). Note that EGF also failed to increase P-TBK1 in HEK293 cells (see (13)). These results demonstrate that EGF does not activate TBK1, at least in MEFs and HEK293 cells. Thus, basal rather than EGF-stimulated TBK1 activity supports mTORC1/2 signaling.

### TBK1 phosphorylates mTOR within mTORC2, interacts with mTORC2, and increases mTORC2 intrinsic catalytic activity

**Figure 5:**
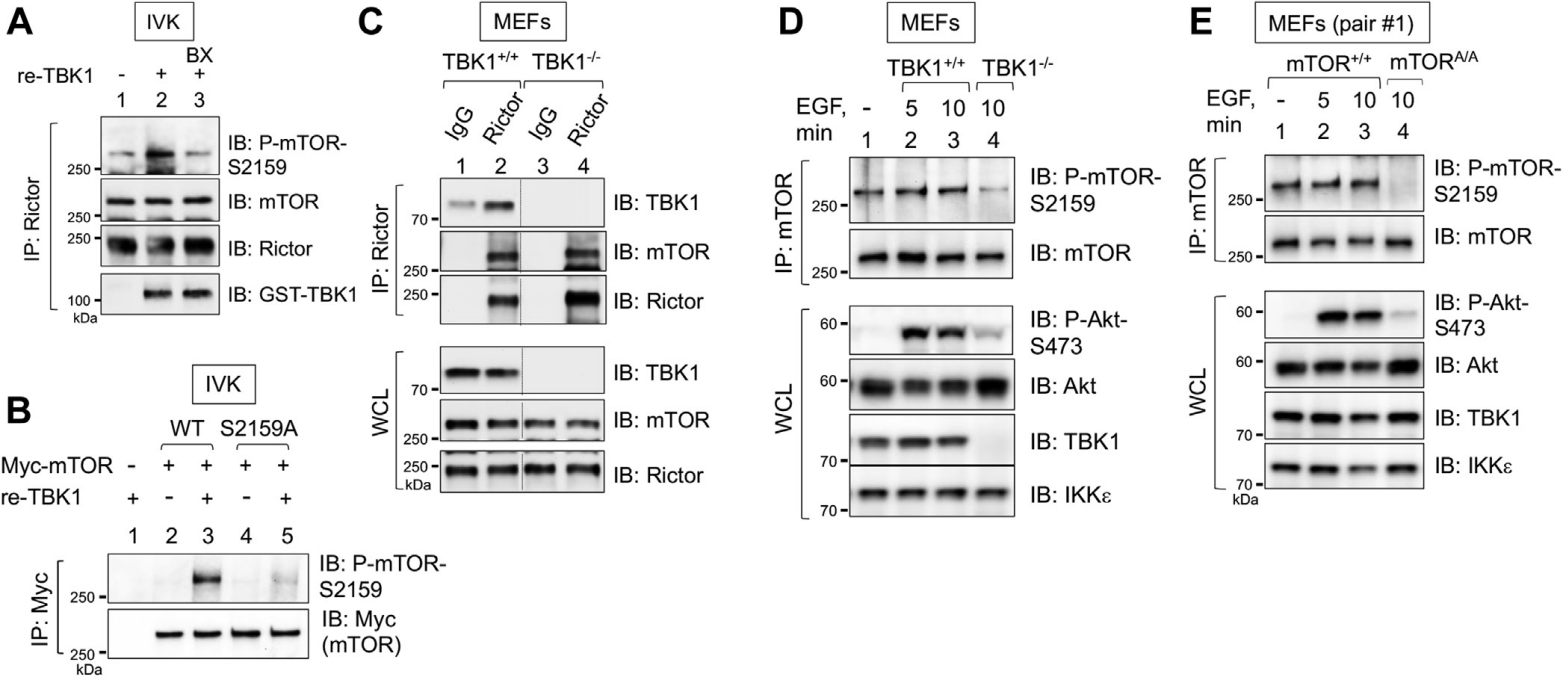
**TBK1 phosphorylates mTOR within mTORC2 and interacts with mTORC2.***A*, TBK1 phosphorylates mTOR S2159 within mTORC2 *in vitro*. Rictor was immunoprecipitated from HEK293 cells and incubated with recombinant, active TBK1 (re-TBK1; 100 ng) for 30 min at 30 °C. The IVK reaction in lane 3 was pretreated with BX-795 (BX; 10 μM) for 30 min as indicated. *B*, recombinant TBK1 phosphorylates Myc-mTOR wildtype but not S2159A *in vitro*. HEK293 cells were transfected with vector control (−), Myc-mTOR wildtype (WT), or Myc-mTOR S2159A. mTOR was immunoprecipitated (IP) with Myc-9E10 antibody and subjected to IVK reactions with re-TBK1 (50 ng) per reaction, as in Figure 2, *D* and *E*. IVK reactions were immunoblotted with the indicated antibodies. *C*, TBK1 coimmunoprecipitates with Rictor and mTOR. Whole-cell lysates (WCLs) from TBK1^+/+^ and TBK1^−/−^ MEFs cultured in complete media (DMEM/FBS) were incubated with Sepharose beads conjugated to either control IgG or anti-Rictor antibodies overnight at 4 °C. The immunoprecipitates (IPs) and WCLs were immunoblotted with the indicated antibodies. *D*, reduced mTOR S2159 phosphorylation in TBK1 null MEFs. mTOR was immunoprecipitated from TBK1^+/+^ and TBK1^−/−^ MEFs that had been serum starved overnight and stimulated with EGF (25 ng/ml, 10 min). IPs and WCLs were immunoblotted with the indicated antibodies. *E*, mTOR S2159 knock-in MEFs lack mTOR P-S2159. mTOR was immunoprecipitated from mTOR^+/+^ and mTOR^A/A^ MEFs as in *C*. IPs and WCLs immunoblotted with the indicated antibodies. All experiments were performed three times or more.

To further define the mechanism by which TBK1 promotes mTORC2 signaling, we asked whether recombinant TBK1 phosphorylates mTOR S2159 within mTORC2 directly. It is important to note that our prior work demonstrated that TBK1 phosphorylates mTOR S2159 within mTORC1 (13). By *in vitro* kinase assay, we found that recombinant active TBK1 increased mTOR P-S2159 on Rictor-associated mTOR immunoprecipitated from cells, and inclusion of the TBK1/IKKε inhibitor BX-795 *in vitro* blocked this increase (Fig. 5*A*). Moreover, we confirmed that TBK1 phosphorylates wildtype but not S2159A Myc-mTOR immunoprecipitated from transfected cells (Fig. 5*B*), confirming the site specificity of the mTOR P-S2159 antibody, as demonstrated previously (13). We next asked whether TBK1 and mTORC2 interact in cells. By coimmunoprecipitating endogenous proteins, we found that Rictor immunoprecipitates pulled-down TBK1 in TBK1^+/+^ but not TBK1^−/−^ MEFs (Fig. 5*C*). Together, these data support a mechanism whereby TBK1 interacts with mTORC2 and subsequently mediates the direct phosphorylation of mTOR S2159 within mTORC2. We next asked whether EGF increases mTOR S2159 phosphorylation and whether TBK1 promotes mTOR S2159 phosphorylation in intact cells. EGF failed to increase P-S2159 on mTOR immunoprecipitated from wildtype TBK1^+/+^ or mTOR^+/+^ MEFs (Fig. 5, *D* and *E*). Of importance, TBK1 knockout reduced mTOR P-S2159 (Fig. 5*D*), and mTOR P-S2159 was undetectable in mTOR^A/A^ MEFs (Fig. 5*E*), confirming *Mtor S2159A* knock-in. We speculate that the remaining mTOR P-S2159 found in TBK1^−/−^ MEFs results from IKKε-mediated phosphorylation of mTOR. Indeed, although IKKε expression is generally tissue restricted and extremely low in nonimmune cells, MEFs express detectable levels of IKKe (Fig. 5, *D* and *E*). These data demonstrate that TBK1 mediates mTOR S2159 phosphorylation *in vitro* and in intact cells and support the conclusion that basal rather than EGF stimulated TBK1 kinase activity mediates mTOR P-S2159 to promote mTORC2 signaling.

**Figure 6:**
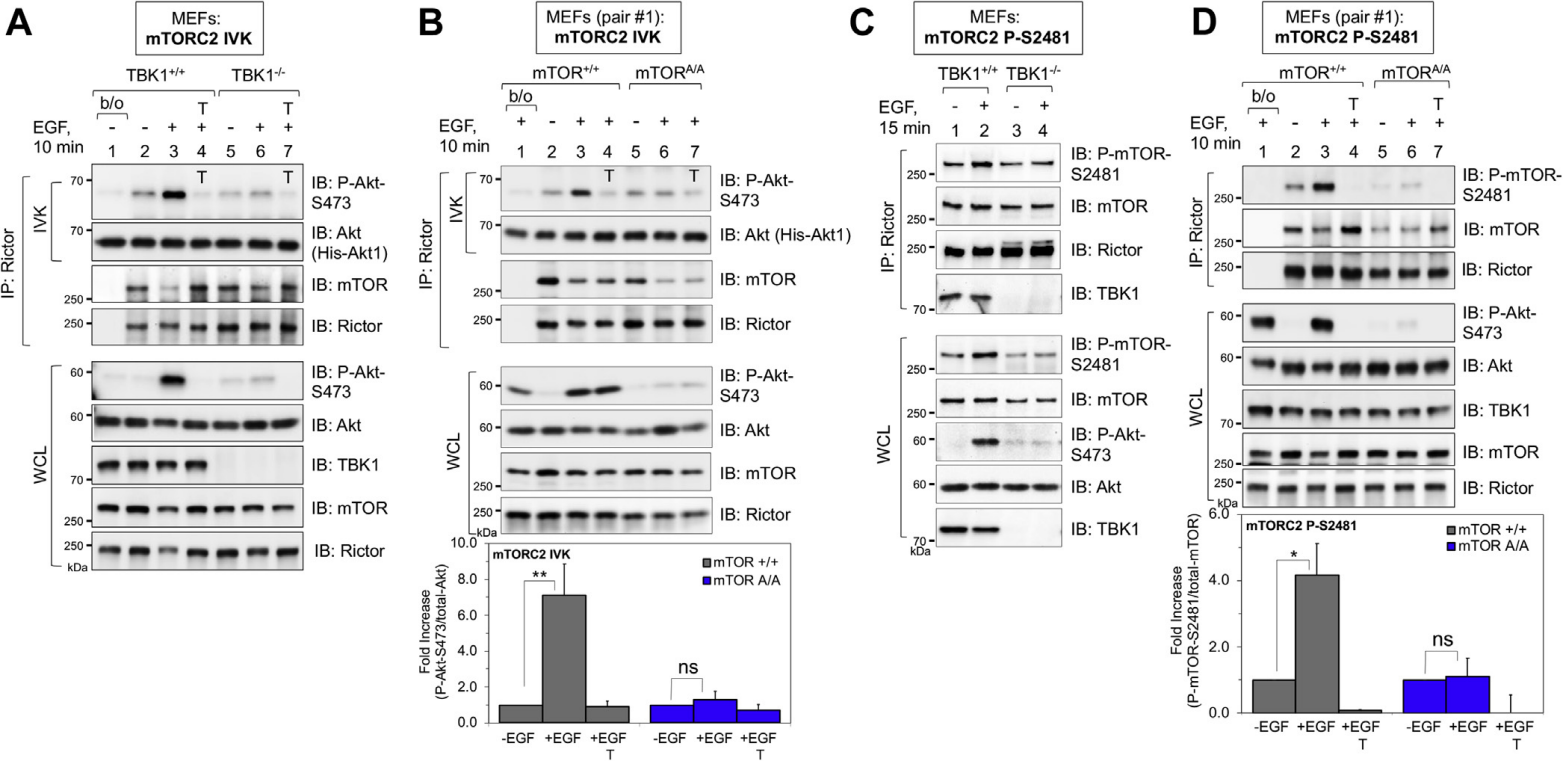
**TBK1 and mTOR S2159 phosphorylation increase mTORC2 catalytic activity.***A*, TBK1 is required for mTORC2 intrinsic catalytic activity. Rictor was immunoprecipitated (IP) from TBK1^+/+^ and TBK1^−/−^ MEFs that had been serum starved overnight, pretreated with Torin1 (100 nM, 30 min), and stimulated with EGF (50 ng/ml, 10 min). The immune complexes were washed and subjected to *in vitro* kinase (IVK) reactions with His-Akt1 (100 ng) substrate and ATP (500 μM) at 30 °C for 30 min. Torin1 was included in the IVK reactions from cells pretreated with Torin1, as indicated (T on the blot). IVKs and whole-cell lysates (WCLs) were immunoblotted with the indicated antibodies. *B*, mTOR P-S2159 is required for mTORC2 intrinsic catalytic activity. Rictor was immunoprecipitated from mTOR^+/+^ and mTOR^A/A^ MEFs (pair #1) that had been treated with EGF. IVK reactions were performed on the immune complexes and analyzed as in *A*, except that only certain IVK reactions (not the cells) were treated with Torin1, as indicated (T on the blot). *Graph*, quantification of results. Mean ratio ± SEM of the fold increase in Akt P-S473 over total-Akt1 from four independent experiments, normalized within each genotype, setting the −EGF condition to 1.0. Statistical significance was measured using paired Student’s *t* test (assuming equal variances). ∗∗*p* < 0.01 relative to unstimulated mTOR^+/+^ MEFs; “ns”, not significant. *C*, TBK1 is required for mTOR S2481 autophosphorylation within mTORC2. Rictor was immunoprecipitated from TBK1^+/+^ and TBK1^−/−^ MEFs that had been serum starved overnight and stimulated with EGF (50 ng/ml, 15 min) as in *A*. IPs and WCLs were immunoblotted with the indicated antibodies. *D*, mTOR P-S2159 is required for mTOR S2481 autophosphorylation within mTORC2. Rictor was immunoprecipitated from mTOR^+/+^ and mTOR^A/A^ MEFs (pair #1) that had been treated with EGF and Torin1 as in *A*. IPs and WCLs were immunoblotted with the indicated antibodies. *Graph*, quantification of results. Mean ratio ± SEM of the fold increase in mTOR P-S2481 over total-mTOR from three independent experiments. ∗*p* < 0.05 relative to unstimulated mTOR^+/+^ MEFs stimulated +EGF; “ns”, not significant. All experiments were performed three times or more.

We next asked whether TBK1 and mTOR S2159 phosphorylation increase the intrinsic catalytic activity of mTORC2 by *in vitro* kinase assays. To do so, we immunoprecipitated Rictor from TBK1^+/+^ *versus* TBK1^−/−^ MEFs and from mTOR^+/+^ *versus* mTOR^A/A^ MEFs after EGF stimulation of serum-deprived cells. The Rictor immunoprecipitates were washed and incubated in kinase buffer with ATP and recombinant His-Akt1 as substrate, and the ability of Rictor-associated mTOR to phosphorylate Akt S473 *in vitro* was monitored by Western blot. In both TBK1^+/+^ MEFs (Fig. 6*A*) and mTOR^+/+^ MEFs (Fig. 6*B*), EGF increased mTORC2 catalytic activity in a Torin1-sensitive manner, as expected. The fold increase in mTORC2 catalytic activity mediated by EGF, however, was reduced in TBK1^−/−^ MEFs (Fig. 6*A*) and mTOR^A/A^ MEFs (Fig. 6*B*). To assess the role of TBK1 and mTOR S2159 phosphorylation in control of mTORC2 catalytic activity by an independent approach, we monitored S2481 autophosphorylation on Rictor-associated mTOR (*i.e.*, mTORC2). Like results obtained with mTORC2 *in vitro* kinase assays, the fold increase in mTOR S2481 autophosphorylation mediated by EGF was reduced in TBK1^−/−^ (Fig. 6*C*) and mTOR^A/A^ MEFs (Fig. 6*D*). Taken together, these results indicate that TBK1 and mTOR S2159 phosphorylation increase mTORC2 catalytic activity.

### TBK1 and mTOR S2159 phosphorylation increase mTORC2 (and mTORC1) signaling in response to diverse growth factors

**Figure 7:**
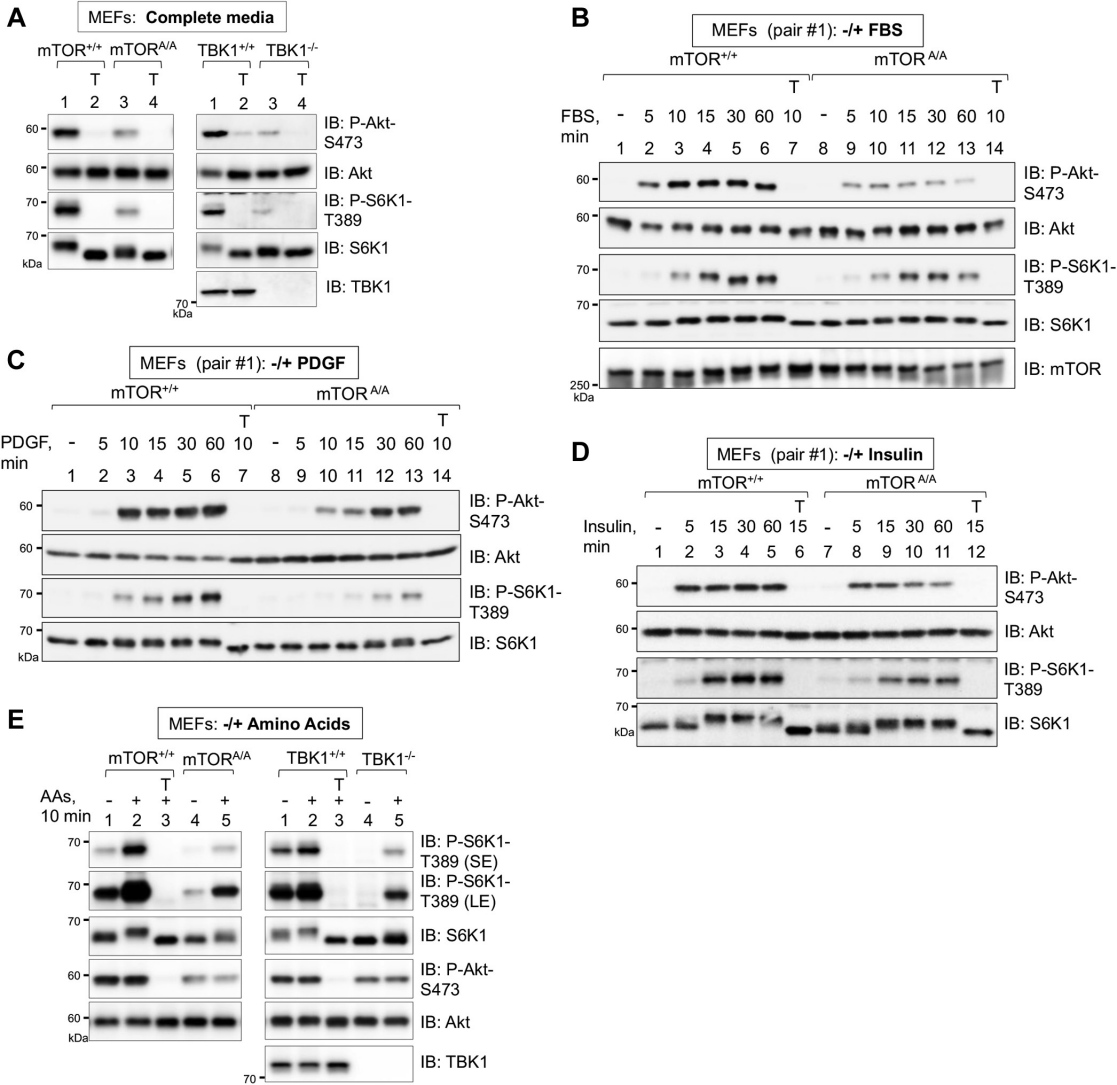
**TBK1 and mTOR S2159 phosphorylation promote mTORC2 and mTORC1 signaling in response to diverse growth factors.***A*, reduced mTORC2 and mTORC1 signaling in mTOR S2159A knock-in MEFs and TBK1 null MEFs in complete media. mTOR^+/+^*versus* mTOR^A/A^ MEFs (pair #1) and TBK1^+/+^*versus* TBK1^−/−^ MEFs were cultured in complete media (DMEM/FBS [10%]). At ∼80% confluency, cells were re-fed with complete media for 1.5 h, treated without or with Torin1 (T) (100 nM, 30 min), and lysed. Whole-cell lysates (WCLs) were immunoblotted with the indicated antibodies. *B*, reduced mTORC2 and mTORC1 signaling in mTOR S2159A knock-in MEFs in response to FBS. mTOR^+/+^ and mTOR^A/A^ MEFs (pair #1) were serum starved overnight (20 h), pretreated with Torin1 (T) (100 nM, 30 min), and stimulated without (−) or with FBS (10% final) for the indicated times. WCLs were immunoblotted with the indicated antibodies. *C*, reduced mTORC2 and mTORC1 signaling in mTOR S2159A knock-in MEFs in response to PDGF. mTOR^+/+^ and mTOR^A/A^ MEFs (pair #1) were serum starved overnight and treated as in *B*, except they were stimulated with PDGF (10 ng/ml). WCLs were immunoblotted with the indicated antibodies. *D*, reduced mTORC2 and mTORC1 signaling in mTOR S2159A knock-in MEFs in response to insulin. mTOR^+/+^ and mTOR^A/A^ MEFs (pair #1) were serum starved overnight and treated as in *B*, except they were stimulated with insulin (100 nM). WCLs were immunoblotted with the indicated antibodies. *E*, mTOR S2159A knock-in and TBK1 knockout reduces mTORC2 and mTORC1 signaling in both the absence and presence of amino acids. mTOR^+/+^*versus* mTOR^A/A^ MEFs (pair #1) and TBK1^+/+^*versus* TBK1^−/−^ MEFs cultured in complete media (DMEM/FBS) were amino acid deprived in DMEM lacking all amino acids but containing 10% dialyzed FBS (dFBS) (50 min). MEFs were then stimulated with a mixture of 1× total amino acids (pH 7.4) (10 min). WCLs were immunoblotted with the indicated antibodies. All experiments were performed three times or more. LE, long exposure; SE, short exposure.

We next investigated whether the positive role of mTOR S2159 phosphorylation in mTORC2 signaling extends to a broader set of growth factors beyond EGF. We thus interrogated mTORC2 signaling to Akt, as well as mTORC1 signaling to S6K1, in mTOR^+/+^ *versus* mTOR^A/A^ MEFs in response to several growth factors known to activate mTORC2 and 1 signaling. We first assessed the role of mTOR P-S2159 in control of mTORC2 and 1 signaling in MEFs cultured in complete media (*i.e.*, Dulbecco's modified Eagle's medium [DMEM]/FBS) containing a full repertoire of serum growth factors. mTOR^A/A^ MEFs as well as TBK1^−/−^ MEFs displayed reduced Akt S473 and S6K1 T389 phosphorylation (Fig. 7*A*). Treatment of cells with Torin1 ablated these phosphorylation events, indicating that mTOR activity is required for mTORC2 and 1 signaling in complete media (Fig. 7*A*). We next assessed the role of mTOR P-S2159 in control of mTORC2 and 1 signaling in response to stimulation of MEFs with fetal bovine serum (FBS), PDGF (a major constituent of FBS), and insulin (which acts through IGF-1 receptors in MEFs) following serum deprivation. Like their response to EGF, mTOR^A/A^ MEFs displayed reduced Akt P-S473 and S6K1 P-T389 in response to all three growth factors with varying dynamics across a time course (Fig. 7, *B*–*D*). In addition, all three growth factors failed to increase TBK1 S172 phosphorylation (Fig. S3), suggesting that growth factor receptor signaling does not activate TBK1, at least in MEFs. These results indicate that mTOR S2159 phosphorylation increases mTORC2 and 1 activity in parallel to growth factor signaling.

Considerable evidence indicates that amino acids increase mTORC1 but not mTORC2 signaling through a mechanism involving recruitment of mTORC1 to the surface of lysosomal membranes, where Rheb resides (30, 61, 62, 63, 64, 65, 66). Curiously, TBK1 knockout reduced Akt S473 phosphorylation when amino acids were added back to amino acid–starved MEFs (14). We therefore examined a role for mTOR P-S2159 in control of mTORC2 signaling in both the absence and presence of amino acids. We cultured mTOR^+/+^ *versus* mTOR^A/A^ MEFs as well as TBK1^+/+^ *versus* TBK1^−/−^ MEFs in DMEM lacking amino acids but supplemented with dialyzed FBS for 50 min. We then added back amino acids to 1× using a 50× amino acid solution whose pH was adjusted to pH 7.4 (from a basic pH of ∼10) for 10 min. We found that overall Akt P-S473 and S6K1 P-T389 was reduced in mTOR^A/A^ and TBK1^−/−^ MEFs in both the absence and presence of amino acids (Fig. 7*E*). As expected, amino acid stimulation increased S6K1 P-T389 but not Akt P-S473 in wildtype, mTOR^A/A^, and TBK1^−/−^ MEFs (Fig. 7*E*), confirming that amino acid stimulation increases mTORC1 but not mTORC2 signaling. These results indicate that TBK1 and mTOR P-S2159 support both mTORC1 and 2 signaling in parallel to growth factor and amino acid signaling pathways.

### TBK1 activity and mTOR S2159 phosphorylation increase TLR3-mediated mTORC2 signaling in macrophages

**Figure 8:**
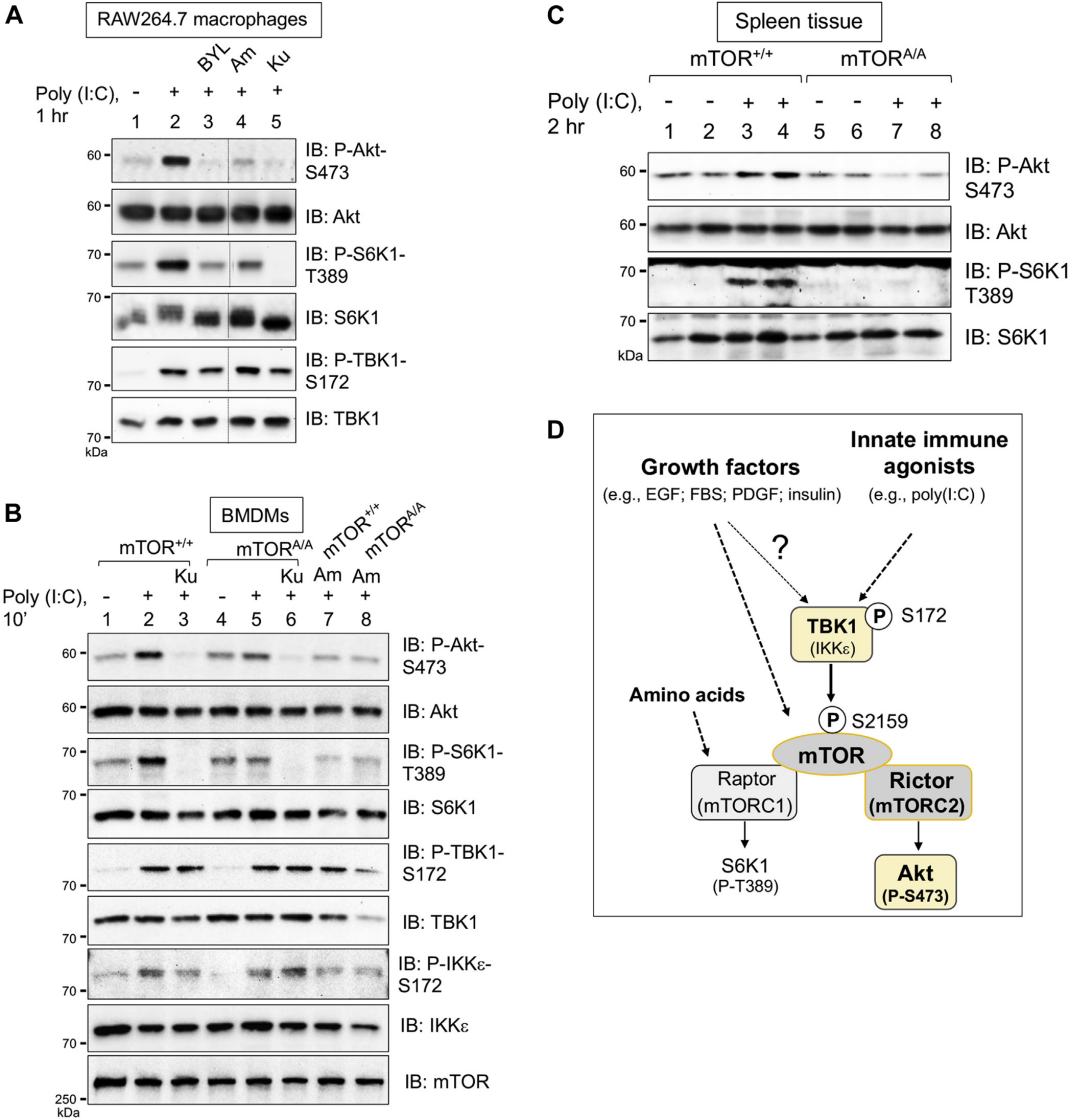
**TBK1 activity and mTOR S2159 phosphorylation increase TLR3-mediated mTORC2 signaling in macrophages.***A*, inhibition of PI3K, TBK1, and mTOR reduces mTORC2 signaling in RAW264.7 macrophages in response to poly(I:C). RAW264.7 macrophages cultured in complete media (DMEM/FBS) were pretreated with BYL-719 (10 μM, 30 min), amlexanox (100 μM, 1 h), or Ku-0063794 (100 nM, 30 min) and stimulated without (−) or with (+) poly(I:C) (30 μg/ml, 60 min). Whole-cell lysates (WCLs) were immunoblotted with the indicated antibodies. *B*, mTOR S2159A knock-in or amlexanox reduces mTORC2 signaling in primary BMDMs in response to poly(I:C). Primary bone marrow–derived macrophages (BMDMs) derived from *Mtor*^*+/+*^ and *Mtor*^*A/A*^ mice were cultured in complete media (DMEM/FBS), pretreated with Ku-0063794 (100 nM, 30 min) or amlexanox (100 μM, 1 h), and stimulated without (−) or with (+) poly(I:C) (30 μg/ml, 10 min). WCLs were immunoblotted with the indicated antibodies. *C*, mTOR S2159A knock-in reduces mTORC2 signaling in mouse spleen tissue in response to poly(I:C) treatment *in vivo*. *Mtor*^*+/+*^ and *Mtor*^*A/A*^ mice were fasted 5 h and injected intraperitoneally with poly(I:C) (10 mg/kg-BW, 2 h). Spleen tissue was isolated, homogenized, and analyzed by Western blotting with the indicated antibodies. *D*, model. See text for details. All experiments were performed three times or more.

Our prior work demonstrated that TBK1 and mTOR S2159 phosphorylation promote mTORC1 signaling in macrophages upon activation of TLR3 and 4, pathogen recognition receptors that activate TBK1 and IKKε (13). To investigate a role for TBK1 and mTOR P-S2159 in positive control of mTORC2 signaling in macrophages, we assessed how TBK1/IKKε inhibitors or *Mtor S2159A* knock-in modulated mTORC2 signaling to Akt in response to TLR3 activation with poly(I:C) (a viral dsRNA mimetic). In cultured RAW264.7 macrophages, poly(I:C) activated TBK1 (as monitored by increased TBK1 P-S172) and PI3K-dependent mTORC2 signaling (as monitored by the sensitivity of Akt P-S473 to the class I PI3K inhibitor BYL-719 and the mTOR inhibitor Ku-0063794 [Fig. 8*A* and Fig. S4]). Inhibition of TBK1/IKKε with two different small molecules, amlexanox (Fig. 8*A* and Fig. S4) or BX-795 (Fig. S4), also reduced poly(I:C)-induced Akt P-S473, demonstrating that TBK1/IKKε activity positively controls mTORC2 signaling to Akt in RAW264.7 macrophages.

We next isolated primary bone marrow derived macrophages (BMDMs) from *Mtor*^*+/+*^ and *Mtor*^*A/A*^ mice. As expected, poly(I:C) increased TBK1 P-S172 and mTOR-dependent Akt P-S473 in wildtype BMDMs (Fig. 8*B*). As in RAW264.7 macrophages, amlexanox suppressed Akt P-473 to a basal level (Fig. 8*B*). Of importance, BMDMs from *Mtor*^*A/A*^ mice displayed reduced poly(I:C)-induced Akt P-S473 (Fig. 8*B*), thus demonstrating a required role for mTOR S2159 phosphorylation in TLR3-mediated activation of mTORC2 signaling. Consistent with our prior work (13), amlexanox or *Mtor S2159A* knock-in reduced mTORC1 signaling in RAW264.7 macrophages and BMDMs (Fig. 8, *A* and *B*). These results demonstrate that TBK1 activity, mTOR activity, and mTOR S2159 phosphorylation are required for mTORC2 signaling to Akt upon activation of TLR3 in macrophages. Finally, to demonstrate a role for mTOR S2159 phosphorylation in activation of mTORC2 signaling by TLR3 *in vivo*, we injected *Mtor*^*+/+*^ and *Mtor*^*A/A*^ mice with poly(I:C) and harvested macrophage-rich spleen tissue. We found that spleen tissue from *Mtor*^*A/A*^ mice displayed reduced Akt S473 phosphorylation in response to poly(I:C) (Fig. 8*C*).

Taken together, these results demonstrate that TBK1 phosphorylates mTOR S2159 to activate mTORC2 directly and thus increase downstream signaling to Akt in cell lines, primary macrophages, and *in vivo* (Fig. 8*D*). Moreover, they reveal that mTORC2 represents an essential link between TBK1 and Akt phosphorylation at physiological levels of protein expression. We find that, in MEFs, basal TBK1 kinase activity signals in parallel to growth factors and amino acids to augment mTORC2 (and mTORC1) activity, as EGF and other growth factors increased mTORC1/2 signaling in a TBK1- and mTOR P-S2159-dependent manner without increasing TBK1 S172 or mTOR S2159 phosphorylation. The relationship between growth factor signaling and TBK1 activity appears to be complex and context dependent, however, as growth factors were shown recently to activate TBK1 (*i.e.*, increase TBK1 P-S172) in lung cancer cells (15) (see Discussion). In macrophages, TLR3 signaling increases TBK1 and mTORC1/2 activity in a linear pathway (Fig. 8*D*). It is important to note that our prior work demonstrated that poly (I:C) increases mTOR-S2159 phosphorylation in RAW264.7 macrophages and primary BMDMs in a manner sensitive to the TBK1/IKKε inhibitor BX-795 (13).

## Discussion

Studies from several groups demonstrated a positive role for TBK1 in Akt phosphorylation in various contexts. Increasing or decreasing TBK1 activity by various approaches in many cell types (*e.g.*, MEFs, HEK293 cells, U2OS cells, HeLa cells, MNT1 melanoma cells, or HCT116 colorectal cancer cells) led to correspondingly similar changes in Akt S473 phosphorylation (14, 15, 16, 17). These observations, together with evidence that mTORC2 serves as a major Akt S473 kinase (19, 20, 21, 22) and our prior work that TBK1 directly activates mTORC1 (13), prompted us to investigate whether mTORC2 represents a missing link between TBK1 and Akt phosphorylation.

Although it is challenging to demonstrate definitely that a kinase (*e.g.*, TBK1) phosphorylates a substrate (*e.g.*, mTOR) directly in intact cells rather than indirectly within a complex, several lines of evidence considered together support our model that TBK1 phosphorylates mTOR S2159 directly to increase mTORC2 activity and signaling to Akt (Fig. 8*D*): TBK1 phosphorylates mTOR S2159 within mTORC2 *in vitro*; TBK1 and mTORC2 coimmunoprecipitate; TBK1^−/−^ MEFs display reduced mTOR P-S2159 in intact cells; TBK1^−/−^ MEFs and mTOR^A/A^ MEFs (lacking mTOR P-S2159) display reduced mTOR-dependent Akt P-S473 and Akt P-T308 in response to EGF; TBK1 overexpression increases Akt P-S473 in a largely Torin1-sensitive manner at endogenous levels of Akt; and finally, both TBK1^−/−^ MEFs and mTOR^A/A^ MEFs display reduced mTORC2 intrinsic catalytic activity in response to EGF, as measured by mTORC2 IVK assay and by Rictor-associated mTOR S2481 autophosphorylation. In support of these results in cell lines, we also demonstrate that, in primary macrophages in culture (*i.e.*, BMDMs) and spleen tissue *in vivo*, mTOR S2159 phosphorylation is required for Akt S473 phosphorylation in response to TBK1 activation with poly(I:C). In addition, Zhao *et al.* (50) found that adipocyte-specific knockout of TBK1 in diet-induced obese mice reduced Akt P-S473 in response to insulin in white adipose tissue. Taken together, these results argue that TBK1-mediated mTOR S2159 phosphorylation promotes mTORC2 signaling to Akt.

Several groups identified TBK1 as a direct Akt S473 and T308 kinase (16, 17, 18). We found that at physiological levels of TBK1 and Akt expression, the ability of growth factors or poly(I:C) to increase Akt S473 phosphorylation in a detectable manner required mTOR activity. mTOR inhibition also reduced Akt P-T308, a finding consistent with the observation that Akt S473 phosphorylation promotes and/or stabilizes Akt T308 phosphorylation (19, 35, 39). We found that overexpression of TBK1 increased Akt P-S473, like earlier work (16, 17, 18). This effect was largely dependent on mTOR activity at low to mid doses of TBK1 but displayed modest mTOR independence at higher doses. When both TBK1 and Akt were overexpressed, however, TBK1 increased Akt P-S473 in an mTOR-independent manner. Taken together, these data indicate that, at physiological levels of TBK1 and Akt expression, TBK1 increases Akt phosphorylation through mTORC2.

Pathological or unique physiological contexts may modify mechanisms governing Akt S473 and T308 phosphorylation. For example, tissue-specific knockout of Rictor or mTOR from cardiac or skeletal muscle failed to ablate Akt S473 phosphorylation (an unexpected finding) (17, 67, 68, 69). Even more surprising, mTOR knockout cardiac muscle displayed elevated Akt P-S473 (17, 69). This finding prompted Xie *et al.* (17) to search for alternate Akt S473 kinases. Upon discovering TBK1 as an Akt S473 and T308 kinase, they noted that mTOR knockout cardiac muscle displayed elevated TBK1 expression. Thus, in this context of elevated TBK1 expression, TBK1 may phosphorylate Akt directly. Pathological contexts may also render Akt S473 phosphorylation TBK1 independent. For example, TBK1 inactivation reduced S6K1 T389 but not Akt S473 phosphorylation in response to growth factors in A549 human lung adenocarcinoma cells or primary mouse lung cancer epithelial cells (15). Therefore, we propose that, in oncogenic contexts or other pathological contexts, cells and tissues may rewire signaling and metabolism by upregulating expression of TBK1/IKKε (or other Akt S473 kinases). Such an adaptive response may in turn enable TBK1/IKKε (or other kinases) to directly phosphorylate Akt S473 and/or T308, thus increasing Akt activity in order to mitigate the pathologic insult and improve metabolic homeostasis and/or provide a proliferative or survival advantage.

Mechanisms governing regulation of TBK1 kinase activity in nonimmune cells remain poorly defined. Our results indicate that, in MEFs, growth factors increase mTORC2 signaling in a TBK1- and mTOR P-S2159-dependent manner without increasing TBK1 S172 phosphorylation (*i.e.*, TBK1 activity) or mTOR S2159 phosphorylation. We therefore conclude that, in this context, the basal kinase activity of TBK1 promotes mTORC2 signaling in parallel to growth factors. It is important to note, however, that stimulation of human A549 lung cancer cells and primary mouse lung cancer cells with several different growth factors (*e.g.*, EGF; FBS; insulin) increased TBK1 S172 phosphorylation, which required TBKBP1 (TBK1 binding protein 1), a TBK1 adaptor protein (15). We speculate that differential expression of numerous TBK1 adaptors explains differences in cellular context that underlies regulation of TBK1 activity by growth factors. These adaptors may also differentially control TBK1 subcellular localization and/or TBK1 substrate preference (7, 46, 70, 71).

Taken together, our results identify TBK1 as a direct activator of mTORC2 in physiological contexts, which expands our limited understanding of mTORC2 regulation. In addition, they establish the TBK1-mTORC2 pathway as a potential target for therapeutic intervention to treat cancer and obesity-linked metabolic disorders.

## Experimental procedures

### Materials

General chemicals were from Thermo Fisher or Sigma. Protein A- and G-Sepharose Fast Flow were from GE Healthcare; NP40, Brij35, and CHAPS (3-[(3-cholamidopropyl)-dimethylammonio]-1-propanesulfonate) detergents were from Pierce; cOmplete Protease Inhibitor Cocktail (EDTA-free) tablets were from Millipore Sigma (#11836170001); Immobilon-P polyvinylidene difluoride (PVDF) membrane (0.45 μM) was from Millipore; Bradford Reagent for protein assays was from Bio Rad (#5000001); reagents for enhanced chemiluminescence were from either Alkali Scientific (Bright Star) or Advansta (Western Bright Sirius HRP substrate). Recombinant active GST-TBK1 was from Thermo Fisher/Life Technologies (#PV3504); recombinant His-Akt1 was from EMD Millipore (#14-279); recombinant GST-mTOR_f_ (containing a 30 kDa fragment of mTOR encoding amino acids 2144-2175) was generated as described (13).

### Antibodies

The following antibodies from Cell Signaling Technology (CST) were used in this study (all rabbit polyclonal antibodies, unless otherwise noted): Akt (#9272); Akt P-S473 (#4060; rabbit mAb D9E XP); Akt P-T308 (#4056, rabbit mAb 244F9); TBK1 (#3013; or #3504, rabbit mAb D1B4); TBK1 P-S172 (#5483, rabbit mAb D52C2 XP); S6K1 (#9202); S6K1 P-T389 (#9234, rabbit mAb 108D2; #9206, mouse mAb IA5); mTOR (#2972); MAPK (#9102); MAPK P-T202/Y204 (#4370, rabbit mAb D13.14.4 E XP); GST (#2624; mouse mAb 26H1); IgG-conjugated Sepharose beads (#3423); Rictor-conjugated Sepharose beads (#5379). The following antibodies were from other commercial vendors: mTOR P-S2481 (Millipore Sigma #09-343); Myc (Millipore Sigma #05-419, mouse mAb 9E10); HA.11 (Bio Legend # 901513, mouse mAb 1612B). The following custom, anti-peptide polyclonal antibodies were generated by us in-house with assistance from a commercial vendor: Rictor (amino acids 6–20; Covance; as described (72, 73)); mTOR (amino acids 221–237, Covance; as described (72, 73)); mTOR P-S2159 (Millipore; as described (13)).

### Plasmids

pcDNA3/Flag-TBK1 wildtype (WT) and pcDNA3/Flag-TBK1kinase dead (K38M) were obtained from Dr A. Saltiel (UCSD School of Medicine, Institute for Diabetes and Metabolic Health). pcDNA3/Flag-HA-Akt was obtained from Addgene (#9021). pRK5/Myc-mTOR WT was originally from Dr D. Sabatini (MIT and the Whitehead Institute) and obtained *via* Addgene (#1861). pRK5/Myc-mTOR S2159A was generated *via* site-directed mutagenesis as described (13). pCI/HA-Rictor was from Dr E. Jacinto (Rutgers University).

### Cell culture, transfection, and drug treatments

All cell lines used in this study (*i.e.*, MEFs, HEK293, RAW264.7 murine macrophages, and primary mouse BMDMs) were cultured in DMEM that contained high glucose (4.5 g/l), glutamine (584 mg/l), and sodium pyruvate (110 mg/l) (Thermo Fisher/Life Technologies) supplemented with 10% FBS (Gibco/Invitrogen). Note that heat-inactivated FBS was used to culture the RAW264.7 macrophages and BMDMs. Cells were incubated at 37 °C in a humidified atmosphere containing 5% CO_2_. HEK293 cells (from ATCC) were transfected using Mirus Trans-It LT1 in accordance with manufacturer’s instructions and lysed ∼24 to 48 h post transfection. To stimulate cells with growth factors, MEFs and HEK293 cells were first serum starved *via* incubation in DMEM containing 20 mM Hepes pH 7.2 overnight, ∼20 h. The cells were then stimulated with EGF (25 or 50 ng/ml) (Sigma Aldrich #E9644 and #E4127), FBS (10% final), PDGF (10 ng/ml) (EMD Millipore #GF149), or insulin (100 nM) (Thermo Fisher/Life Technologies #12585) for various amounts of time (0–60 min). To stimulate macrophages with innate immune agonists, RAW264.7 macrophages and BMDMs cultured in complete DMEM (*i.e.*, DMEM containing dialyzed FBS [10%]) were treated with poly(I:C) (30 μg/ml) (Sigma Aldrich #P1530) or ultrapure LPS (100 ng/ml) (InVivo Gen #tlrl-3pelps). Cells were treated with the following drugs: Torin1 (100 nM, 30 min) (shared by Dr D. Sabatini), Ku-0063794 (100 nM, 30 min) (Tocris #3725); amlexanox (100 μM, 1–2 h) (EMD Millipore #SML0517); BX-795 (10 μM, 30 min) (Millipore CalBiochem #204011) (30 min); BYL-719 (10 μM, 30 min) (Selleck; #1020). To amino acid deprive and stimulate cells, MEFs were first cultured for 50 min in DMEM lacking all amino acids (US Biologicals #D9800-13) but containing dialyzed FBS (10%) (Life Technologies #A33820-01). The cells were then acutely stimulated with amino acids for 10 min using a 50× solution of total amino acids (RPMI 1640 Amino Acid Solution) (Millipore Sigma; # R7131) supplemented with L-glutamine (Sigma # G8540) to a final concentration of [1×]. Note that the pH of this amino acid solution is quite basic (∼pH 10), and thus the pH of this solution was first normalized to pH 7.4 prior to addition to cells in order to maintain physiological pH.

### Cell lysis, immunoprecipitation, and immunoblotting

Cells were washed twice with ice-cold PBS pH 7.4 and scraped into ice-cold lysis buffer A (10 mM KPO4 pH 7.2; 1 mM EDTA; 5 mM EGTA; 10 mM MgCl2; 50 mM b-glycerophosphate; 1 mM sodium orthovanadate [Na_3_VO_4_]; a cocktail of protease inhibitors) containing NP-40 (0.5%) and Brij35 (0.1%), as described (73). To maintain detergent-sensitive mTOR–partner protein interactions during Rictor or mTOR immunoprecipitation, cells were lysed in ice-cold buffer A containing mild CHAPS [0.3%] detergent. Lysates were spun at 13,200 rpm for 5 min at 4 °C, and the post-nuclear supernatants were collected and incubated on ice (15 min). Bradford assay was used to normalize protein levels for immunoblot or immunoprecipitation analysis. For immunoprecipitation, whole-cell lysates were incubated with antibodies for 2 h at 4 °C, followed by incubation with Protein G- or A-Sepharose beads for 1 h. Sepharose beads were washed three times in lysis buffer and resuspended in 1x sample buffer. Samples were resolved on SDS-PAGE and transferred to PVDF membranes in Towbin transfer buffer containing 0.02% SDS, as described (73). Immunoblotting was performed by blocking PVDF membranes in Tris-buffered saline (TBS) pH 7.5 with 0.1% Tween-20 (TBST) containing 3% nonfat dry milk, as described (73), and incubating the membranes in TBST with 2% bovine serum albumin containing primary antibodies or secondary horseradish peroxidase–conjugated antibodies. Blots were developed by enhanced chemiluminescence and detected digitally with a Chemi-Doc-It System (UVP).

### Lentiviral transduction

TBK1^−/−^ MEFs stably expressing Flag-TBK1 (wildtype or kinase dead K38M) were generated by lentiviral transduction. Flag-TBK1 was subcloned into a modified lentiviral vector, pHAGE-Puro-MCS (pPPM) (74) (modified by Amy Hudson [Medical College of Wisconsin] to include an expanded multiple cloning site). Lentivirus particles were packaged in HEK293T cells by cotransfection with empty pPPM vector or pPPM/Flag-TBK1 together with pRC/Tat, pRC/Rev, pRC/gag-pol, and pMD/VSV-G using Mirus TransIT-LT1 transfection reagent. Supernatants containing viral particles were collected 48 h post transfection and filtered through a 0.45-μm filter. TBK1^−/−^ MEFs were infected with fresh viral supernatants containing 8 μg/ml polybrene. Twenty-four hours post infection, cells were re-fed with DMEM/10% FBS supplemented with 10 μg/ml puromycin for 2 to 3 days to select for stably transduced cells, trypsinized, and plated at limiting dilution in order to isolate clones originating from single cells. TBK1^−/−^ MEF clones transduced with wildtype Flag-TBK1 lentivirus were analyzed for expression of exogenous Flag-TBK1 relative to expression of endogenous TBK1 found in wildtype MEFs. TBK1^−/−^ MEF clones expressing Flag-TBK1 at a level similar to endogenous TBK1 were chosen for analysis. Alternately, TBK1^−/−^ MEFs transduced with wildtype or kinase dead (K38M) Flag-TBK1 were selected in puromycin and pooled for analysis. Rictor ^−/−^ MEFs (from Dr E. Jacinto; Rutgers University) stably expressing vector control or HA-Rictor were generated by lentiviral transduction and stable selection, as described (35). These rescued lines were maintained in DMEM/FBS containing puromycin (8 μg/ml). To knockdown TBK1, Rictor^−/−^ MEFs were infected with lentiviral particles encoding an shRNA targeting TBK1 (Sigma) (mouse TBK1 # TRCN0000323444; nontargeting # SHC016V) and then selected in puromycin (8 μg/ml) for 4 days.

### *In vitro* kinase assays

#### Phosphorylation of recombinant His-Akt1 or GST-mTOR_f_ by recombinant active TBK1

*In vitro* kinase (IVK) assays were performed by incubating recombinant His-Akt1 (50 ng) or GST-mTOR_f_ (50 ng) substrates with recombinant active GST-TBK1 (50 ng) in kinase buffer containing 25 mM Tris-HCl pH 7.5, 10 mM MgCl_2_, 1 mM DTT, and 200 μM ATP. Reactions were incubated at 30 °C for 30 min and stopped by addition of sample buffer followed by incubation at 95 °C for 5 min. Samples were resolved on SDS-PAGE, transferred to PVDF membrane, and immunoblotted. The phosphorylation of His-Akt1 was measured using anti-Akt P-S473 antibodies, and the phosphorylation of GST-mTOR_f_ was monitored using anti-mTOR P-S2159 antibodies. For certain IVK reactions, recombinant kinases were preincubated with BX-795 (10 μM) in kinase buffer on ice for 30 min.

#### Phosphorylation of Myc-mTOR or Rictor-associated mTOR isolated from cells by recombinant active TBK1

HEK293 cells were transfected with Myc-mTOR wildtype or mutant S2159A, and lysates were immunoprecipitated with anti-Myc antibodies. Myc-mTOR S2159A was generated by QuikChange mutagenesis, as described (12). Alternately, Rictor from nontransfected HEK293 cells was immunoprecipitated with anti-Rictor antibodies. After washing in lysis buffer and kinase buffer, the immune complexes (containing substrate) were incubated with recombinant active GST-TBK1 (100 ng) in kinase buffer and ATP, as described above. The phosphorylation of Myc-mTOR or Rictor-associated mTOR was monitored using anti-mTOR P-S2159 antibodies.

#### Phosphorylation of His-Akt1 by cellular mTORC2 (*i.e.*, mTORC2 IVKs)

mTORC2 IVK assays were performed as described (35, 75). Briefly, Rictor was immunoprecipitated from serum-starved MEFs pretreated without or with Torin1 (100 nM, 30 min) and then stimulated without or with EGF (50 ng/ml, 10 min) (∼one 10-cm plate for each immunoprecipitate). After washing in lysis buffer and kinase buffer, the immune complexes (containing mTOR kinase) were incubated with ATP (500 μM) and recombinant His-Akt1 (100 ng/reaction) in kinase buffer containing 25 mM Hepes, 100 mM potassium acetate, and 1 mM MgCl_2_ at 30 °C for 30 min. Certain reactions were also pretreated with Torin1 (10 μM) in kinase buffer on ice for 30 min prior to initiating the IVK reaction.

### Coimmunoprecipitation of TBK1 and mTORC2

TBK1^+/+^ or TBK1^−/−^ MEFs were lysed in buffer A containing CHAPS (0.3%) detergent. Protein levels in each cell type were normalized after performing protein assays with the Bradford assay. IgG control Sepharose beads (CST) or Rictor-conjugated Sepharose beads (CST) were washed 2x with PBS, blocked ∼1 h in PBS containing 2% bovine serum albumin to reduce nonspecific binding, and then washed in PBS 2× more. Whole-cell lysates were then added to the washed beads, rotated at 4 °C overnight, washed 3× in lysis buffer, and resuspended in 1x SDS-PAGE sample buffer.

### Mtor S2159A knock-in mice

mTOR knock-in S2159A mice (*Mtor*^*A/A*^) were generated using CRISPR-Cas9 genome editing technology and genotyped, as described (13). Mice were housed in a specific pathogen-free facility with a 12-h light/12-h dark cycle and given free access to food and water. All animal use was in compliance with the Institutional Animal Care and Use Committee at the University of Michigan.

### Isolation and immortalization of MEFs

Male and female heterozygous *Mtor*^*+/A*^ mice were mated, and MEFs from plugged females were isolated on day 13.5 of pregnancy, generally as described (76, 77). Dissected embryos were washed with 3x in PBS pH 7.4 and minced with fine scissors in the presence of trypsin-EDTA. The minced tissue was triturated with 5 ml serological and fine-tip Pasteur pipettes to prepare a homogenate and taken into a 15-ml conical tube containing 8 ml DMEM. The homogenate was centrifuged 4 min at 300*g*, and the supernatant was discarded. The pellet was washed 1x in PBS pH 7.4 and resuspended in complete medium (DMEM/FBS [10%] containing 50 U/ml penicillin and 50 μg/ml streptomycin). The resuspended cells were transferred to a 10-cm culture dish with fresh complete medium and incubated at 37 °C in a humidified atmosphere containing 5% CO_2_. The MEFs were washed 1× with PBS pH 7.4, detached with 0.05% trypsin-EDTA, centrifuged 4 min at 300*g*, and transferred to a 15-cm culture dish. At confluency, the MEFs were trypsinized and aliquoted to multiple cryovials for long-term storage in liquid N_2_. MEFs were immortalized spontaneously through serial passaging. As they reached confluency in 15-cm culture dishes following isolation, primary MEFs were plated into 10-cm culture dishes at a 1:9 split ratio into 10 ml complete medium (passage #2). Upon reaching confluency, the MEFs were again passaged into 10-cm culture dishes with a split ratio of 1:3. This process was repeated every 3 to 4 days until senescence was reached (∼passage 5–7). The culture medium was replaced every 3 to 4 days, and the cells were trypsinized and transferred into a new culture dish once a week. Once the MEFs began proliferating, they were passaged with a 1:3 split ratio every 3 to 4 days until the cell number doubled every ∼24 to 30 h. MEFs were genotyped as described (13) using dissected head tissue.

### Isolation of primary bone marrow–derived macrophages

Bone marrow from 8- to 14-week-old *Mtor*^*+/+*^ and *Mtor*^*A/A*^ mice was harvested by flushing femora and tibiae with ice-cold PBS pH 7.4 using a 30G needle under sterile conditions. Bone marrow cells were suspended in MEM with L-glutamine supplemented with 10% HI-FBS, 50 U/ml penicillin, 50 μg/ml streptomycin, and 20 ng/ml M-CSF (R&D Systems; #416-ML) and plated into 6-well tissue culture plates. Cells were incubated at 37 °C in 5% CO_2_, and the medium was replaced every other day until day 5, at which point the monocytes had differentiated into macrophages. Macrophages were studied at ∼80% confluency.

### *In vivo* poly(I:C) treatment of mice

*Mtor*^*+/+*^ and *Mtor*^*A/A*^ mice (mostly C57BL6, 12 weeks old) fed a normal chow diet were fasted (5 h) and injected with saline or poly (I:C) (10 mg/kg-BW, 2 h). Spleen tissue was isolated, homogenized, and analyzed by Western blotting. A motorized tissue homogenizer (Tissue Ruptor, Qiagen) was used to homogenize whole spleen in 1 ml ice-cold RIPA buffer that contained protease and phosphatase inhibitors. The lysate was incubated on ice (10 min) and centrifuged at 13,200 rpm (15 min, 4 °C), and the supernatant was collected. DC (Detergent Compatible) protein assay (Bio-Rad; #5000111) was used to standardize protein amount for immunoblot analysis.

### Western blot editing, quantification, and statistical analysis

Western blot images were prepared for publication using Adobe Photoshop. Only the parameters levels, brightness, and contrast were employed to fine-tune exposure time and band sharpness. Of importance, these parameters were adjusted equivalently across the entire blot, and the final image shown reflects the raw image. In certain panels, thin dotted lines indicate excision of an irrelevant lane(s) from a Western blot. Western blot signals were quantitated using FIJI to measure integrated densities of protein bands. The ratios of phosphorylated proteins over cognate total protein were calculated and normalized as indicated in the figure legends. Statistical significance was tested using paired Student’s *t* test assuming equal variances. Error bars represent either standard deviation (SD) or standard error of the mean (SEM), as indicated in the figure legend.

## Data availability

All data are contained in this article.

## Supporting information

This article contains supporting information.

## Conflict of interest

The authors declare that they have no conflicts of interest with the contents of this article.
