# Supplementary material for: The innate immune kinase TBK1 directly increases mTORC2 activity and downstream signaling to Akt
Source: J Biol Chem. 2021 Jul 8;297(2):100942. doi: 10.1016/j.jbc.2021.100942 (PMC8342794; doi:10.1016/j.jbc.2021.100942)

Supplementary Figures

Figure S1 (related to Figure 1)
TBK1 knockout reduces EGF stimulated Akt T308 phosphorylation. $TBK1^{+/+}$ and $TBK1^{-/-}$ MEFs were serum starved overnight (20 hr), pre-treated with Ku-0063794 (Ku) [100nM] (30 min), and stimulated without (-) or with (+) EGF [50 ng/mL] for the indicated times. Whole cell lysates were immunoblotted with the indicated antibodies. All experiments were performed three times or more.

Figure S2 (related to Figure 4)
S2A. mTOR S2159 knock-in reduces Akt T308 phosphorylation in response to EGF. Immortalized $mTOR^{+/+}$ and $mTOR^{A/A}$ MEFs (pair #1) were serum starved overnight (20 hr), pre-treated with Torin1 (T) [100nM] (30 min), and stimulated without (-) or with (+) EGF [50 ng/mL] for the times indicated. Whole cell lysates (WCLs) were immunoblotted with the indicated antibodies.
S2B. EGF fails to modulate TBK1 P-S172 in $TBK1^{+/+}$ MEFs. $TBK1^{+/+}$ and $TBK1^{-/-}$ MEFs were serum starved overnight and stimulated with EGF for the times indicated, as in S2A. RAW264.7 macrophages in complete media were stimulated without (-) or with LPS [100 ng/mL] (1 hr) to serve as a positive control for TBK1 P-S172 western blotting. WCLs from MEFs and RAW264.7 macrophages were resolved on the same gel and immunoblotted with the indicated antibodies. All experiments were performed three times or more.

Figure S3 (related to Figure 7)
Diverse growth factors fail to modulate TBK1 P-S172 in $mTOR^{+/+}$ MEFs.
$mTOR^{+/+}$ and $mTOR^{A/A}$ MEFs (pair #1) were serum starved overnight and stimulated without (-) or with (+) FBS [10%], PDGF [10 ng/mL], or insulin [100 nM] (10 min). RAW264.7 macrophages in complete media were stimulated without (-) or with (+) LPS [100 ng/mL] (1 hr) to serve as a positive control for TBK1 P-S172 western blotting. Whole cell lysates from MEFs and RAW264.7 macrophages were resolved on the same gel and immunoblotted with the indicated antibodies. Note that protein amounts were not normalized between the two cell types. All experiments were performed three times or more.

Figure S4 (related to Figure 8)
Effect of the TBK1/IKKe inhibitor BX-795 on mTORC2 signaling in RAW264.7 macrophages in response to poly(I:C). RAW264.7 macrophages cultured in complete media (DMEM/FBS) were pre-treated with the TBK1/IKKε inhibitor BX-795 (BX) [10 mM] (30 min) as well as amlexanox (Am) [100 μM] (1 hr) and Ku-0063794 (Ku) [100 nM] (30 min) and stimulated without (-) or with (+) poly(I:C) [30 μg/mL] (60 min). Whole cell lysates were immunoblotted with the indicated antibodies. All experiments were performed three times or more.

Supplementary Figure S1 (related to Figure 1)

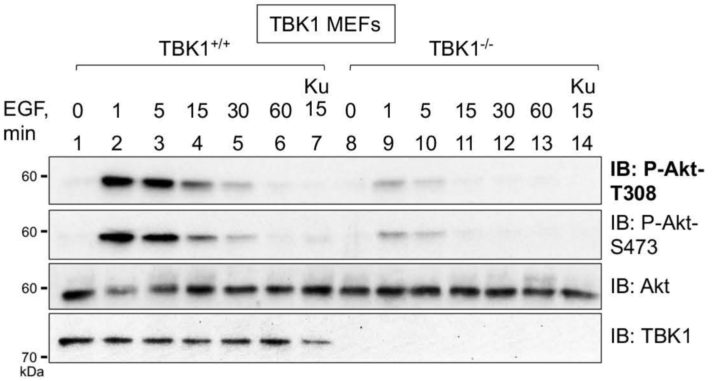

Supplementary Figure S2
(related to Figure 4)

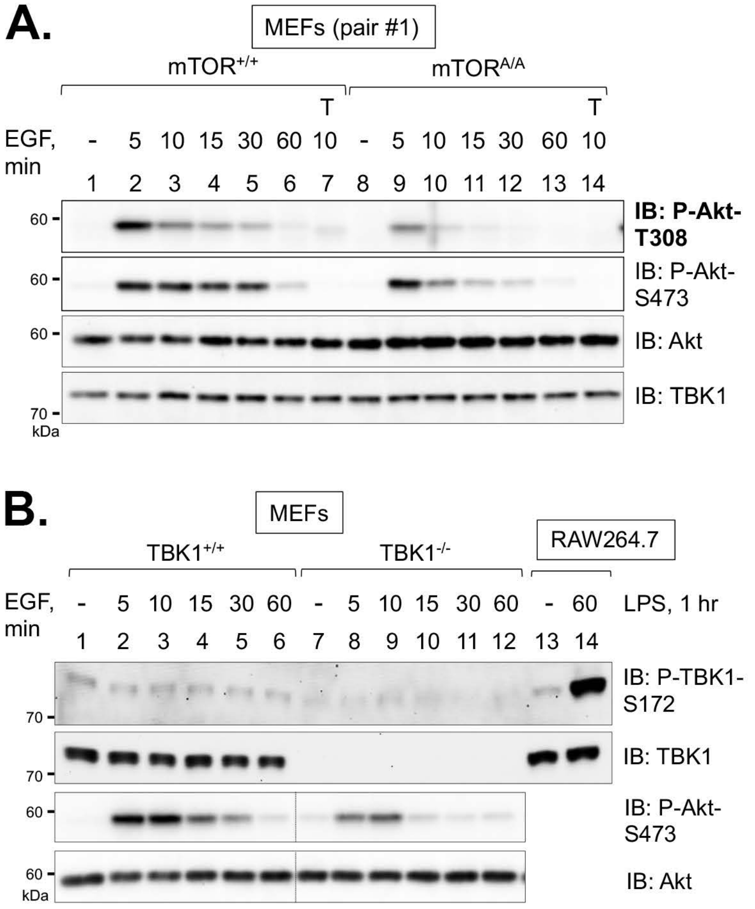

Supplementary Figure S3 (related to Figure 7)

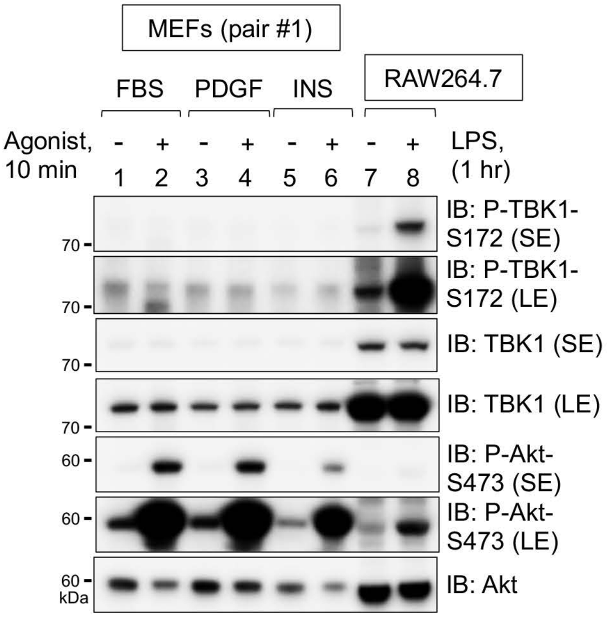

Supplementary Figure S4
(related to Figure 8)

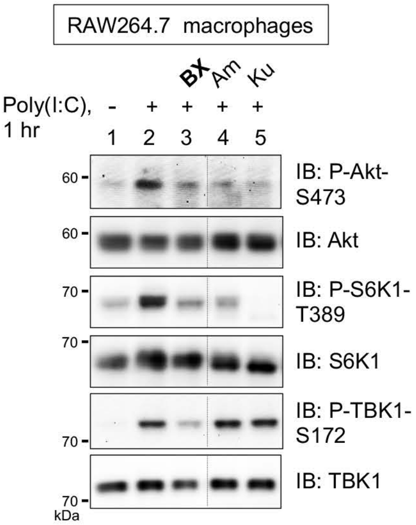

Supplement: Supplemental Figures S1–S4 [file mmc1.pdf]
